# Physicochemical water quality in coastal marine ecosystems: spatiotemporal variation between protected and disturbed areas

**DOI:** 10.7717/peerj.20855

**Published:** 2026-03-19

**Authors:** Isaac Manuel Romero Borja, Rocio García-Urueña, Aliano José Tette Pomárico

**Affiliations:** Grupo de Investigación Ecología y Diversidad de Algas Marinas y Arrecifes Coralinos, Universidad del Magdalena, Santa Marta, Magdalena, Colombia

**Keywords:** Spatiotemporal dynamics, Physicochemical variables, Water quality, Water discharge, Coastal pollution

## Abstract

The monitoring of the continuous fluctuation of seawater environmental conditions plays a critical role in the management and conservation of marine and coastal ecosystems. Although previous studies in the Santa Marta region (Colombian Caribbean) have examined aspects of seawater quality, the spatiotemporal dynamics of key water quality parameters, impact gradients, and contaminant dispersion remain poorly characterized. This study presents the results of a 14-month monitoring program conducted from 2021 to 2024 at seven sampling stations: four in Santa Marta Bay and three within Tayrona National Natural Park (TNNP). These stations cover areas experiencing varying degrees of anthropogenic impact. The assessment of spatiotemporal dynamics of physicochemical seawater parameters revealed seasonal and anthropogenic influences on water quality. During the rainy periods, increased levels of turbidity, nutrients, pollutants, and oxygen demand were observed, particularly at stations impacted by wastewater discharges. Overall, these parameters exceeded the permissible limit frequency during the rainy periods, where 60% of the samples showed elevated levels of organic load. This revealed an eutrophication gradient extending from the most impacted stations to the sites located within the TNNP. Water quality index (Integrated Coastal Area Management; ICAM) assessments confirmed degraded conditions near sewage, river, and port discharge points, while TNNP stations exhibited acceptable environmental quality. These findings emphasize the protective role of marine protected areas as buffers against pollution, and highlight the need for periodic, long-term monitoring to elucidate links between water quality and ecosystem health, critical for informing conservation and management efforts in regions facing substantial anthropogenic pressures such as Santa Marta Bay and the TNNP.

## Introduction

Coastal areas are important for recreation and tourism and are among the main economic sources in port regions. However, these environments are increasingly threatened by global pressures such as climate change, sea-level rise, and ocean acidification (Trégarot et al., 2024), as well as local stressors including erosion from hurricanes and tropical cyclones, pollution, population growth, urban expansion, and overexploitation of natural resources (Drius et al., 2019; De Giglio et al., 2022). Urban runoff, land-based discharges, and treated or untreated wastewater introduce significant loads of exogenous materials to coastal waters (Witt, Wild & Uthicke, 2012; Browne et al., 2014; Landrigan et al., 2020; Rogers & Ramos-Scharrón, 2022). Urban and industrial activities in these areas are major source of organic pollutants such as fats, oils, hydrocarbons, and aromatic compounds (Tosic et al., 2019). In addition, intensive agricultural practices contribute to nutrient loading through nitrogen and phosphorus-reache fertilizers that reached into local river systems, ultimately discharging ammonia, nitrate, nitrite, and phosphate into marine habitats (INVEMAR, 2020; INVEMAR, 2022; INVEMAR, 2024). The nutrient-enriched waters can promote the proliferation of microorganisms and algae, leading to degradation of ecosystem health (García, Palacio & García, 2012; Salomón, Rivera-Rondón & Zapata, 2020). Moreover, land runoff, river inputs, and wastewater discharge increase the presence of particulate matter and sediments inputs, disrupting biogeochemical cycles, diminishing water quality, and hindering the settlement of benthic organisms (Chahal et al., 2016; García-Aljaro et al., 2019).

The Colombian Caribbean coast, known for its exuberant beauty and natural richness, is a prime destination for tourism. A notable example is Santa Marta Bay, located on the Caribbean Sea, which holds high cultural and economic significance. However, the bay faces persistent water quality challenges due to a deficient sewage system, poor wastewater treatment, and high frequency of maritime traffic, being the second most important port in the country (Moscarella, García & Palacio, 2011; Rangel-Buitrago et al., 2013; Garcés-Ordóñez et al., 2020; Rodríguez-Grimón, Campos & Braga Castro, 2021; Romero-Murillo, Gallego & Leignel, 2023). Santa Marta Bay also receives inputs from multiple pollution sources, including a submarine outfall, local wastewater discharges, and inputs from local rivers such as the Manzanares, Gaira, and Magdalena River, as well as from the Cienaga Grande de Santa Marta (Ramos-Ortega et al., 2008; García, Palacio & García, 2012; INVEMAR, 2024). These conditions vary markedly over space and time, especially during the rainy season and near discharge points, resulting in fluctuations in temperature, salinity, dissolved oxygen, chlorophyll, phosphates, turbidity, and microbial loads (Tosic et al., 2019; Vajravelu et al., 2018; Haber et al., 2022). These parameters are routinely monitored using standard physicochemical techniques (Garay Tinoco, Pinilla González & Díaz Merlano, 2003; Rodríguez-Grimón, Campos & Braga Castro, 2021; Braga et al., 2022). However, their spatiotemporal dynamics remain poorly characterized.

The establishment of Marine Protected Areas (MPAs) along the Colombian Caribbean, such as Tayrona National Natural Park (TNNP) has played a critical role in conserving biodiversity, and safeguarding sensitive habitats, including mangroves, seagrass meadows, soft-bottom , and coral reefs. MPAs help buffer anthropic pressures and maintain ecosystem stability (Abreu et al., 2016; INVEMAR, 2022), mitigating coastal pollution and environmental degradation (Lubchenco et al., 2003; Lester et al., 2009). They serve as reference sites for assessing the impacts of human pressure on marine ecosystems and support the development of evidence-based management strategies (Jung et al., 2014; Lukhabi et al., 2023). However, despite their ecological importance, long-term monitoring of coastal water quality in Colombian remains limited.

With increased environmental disturbances, coastal management programs have recognized the necessity of monitoring microbiological, physical, and chemical indicators of water quality to identify pollution sources and assess ecological impacts (Jung et al., 2014; INVEMAR, 2024; Lukhabi et al., 2023). Understanding the spatial and temporal dynamics of these indicators is fundamental to preserving marine ecosystem health and ensuring water safety for public use (Abreu et al., 2016). In Santa Marta, the Surveillance Network for the Conservation and Protection of Marine and Coastal Waters of Colombia (REDCAM), tracks these variables at 15 coastal marine stations, including six sites at key discharge points (INVEMAR, 2024).

In addition the monitoring network also conducts the assessment of the Coastal and Marine Water Quality Index (ICAM) at specific stations and on a periodic basis. Its importance lies in its ability to identify pollution gradients, diagnose anthropogenic impacts, and support environmental management and decision-making in coastal monitoring programs. ICAM is particularly suitable for Colombian marine-coastal ecosystems because it integrates variables sensitive to natural processes (such as upwelling and seasonal mixing) and human pressures (urban, port, and tourism discharges), providing a holistic view of ecological status and enabling comparisons across different sites and time periods (Castillo-Viana et al., 2022).

Despite previous efforts to assess physicochemical water quality parameters in Santa Marta Bay and the TNNP, these changes have not been integrated with wastewater discharges and precipitation in relation to spatiotemporal dynamics. For example, key physicochemical variables (total suspended solids (TSS), turbidity, biochemical oxygen demand (BOD5), chemical oxygen demand (COD)) and pollutant-related indicators (fats and oils, polycyclic aromatic hydrocarbons (PAHs), nutrients, and fecal indicator bacteria) have not been considered. This lack of integration limits the understanding of temporal fluctuations, impact gradients, and the dispersion of pollutants from discharge points to adjacent coastal areas. This approach over 14 months throughout four years, enhances understanding of pressures on marine ecosystems and supports the development of conservation and management strategies for MPAs, which require monitoring aligned with hydrological dynamics, anthropogenic influences, and climate variability. Strengthening management in the TNNP also depends on coordinated institutional, scientific, and community actions that enable better control of pollutant discharges and the design of ecosystem restoration measures. Ultimately, this research provides essential support for public policy, compliance with environmental regulations, and participatory governance that promotes the sustainable use and conservation of marine biodiversity in the Colombian Caribbean.

## Materials & Methods

### Study area

**Figure 1:**
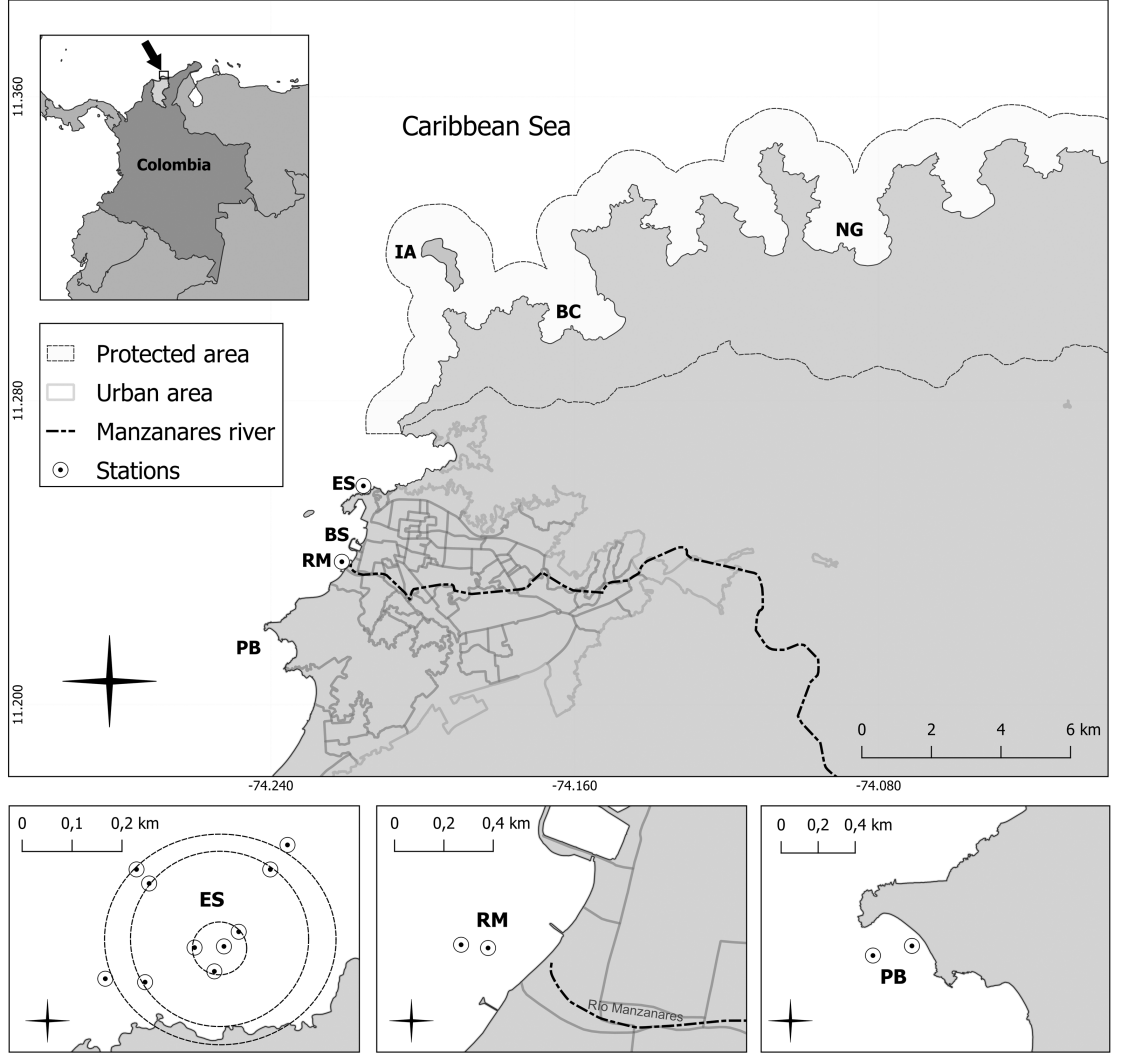
Study area and sampling stations in urban zones (influenced by water discharges) and protected areas within Tayrona National Natural Park (less influenced by discharges), including the Manzanares River.

The coastal area of Santa Marta, Colombian Caribbean is located between 11°13′00″11°15′30″N and between 74°12′30′ and 74°14′30″W (Fig. 1A). It has a narrow rocky continental shelf (Márquez, 1993; Ramos-Ortega et al., 2008), where port and tourist activities predominate. The regional climate is traditionally characterized by two distinct seasons: a dry season (December-April) marked by cold waters (25° to 27 °C) and elevated salinity levels (>36), and a rainy season (May-November), associated with warmer water (27° to 29.5 °C) (Mancera, Pinto & Vilardy, 2013; García-Rentería, Nieto & Cortez, 2023) and reduced salinity (<34) due to increased freshwater input from river discharge and surface runoff (Andrade-Amaya, 1993; Cabrera-Luna & Donoso, 1993; Moscarella, García & Palacio, 2011; Vega-Sequeda et al., 2019).

The Santa Marta coastal zone is subject to multiple sources of anthropogenic discharges. A major contributor is a submarine outfall system composed of wastewater collection tanks connected to a 428-meter-long pipeline with a 1-meter diameter, installed at a depth of 56 m, which that continuously releases approximately 950 L s^−^^1^ of untreated sewage directly into the marine environment. In addition, diffuse discharges occur along the coastline, mainly due to sewer overflows associated with urban areas such as Santa Marta, Taganga, and El Rodadero. Other sources of pollution originate from local rivers, which collect runoff from agricultural and livestock activities along their courses before discharging into the sea (Moscarella, García & Palacio, 2011).

To assess the spatiotemporal dynamics of key physicochemical parameters of water quality along coastal waters of Santa Marta Bay and TNNP, seven sampling stations were established (Fig. 1). These sites represent two contrasting exposure levels to anthropogenic inputs: (1) stations within the TNNP with low influence (or protected) from major discharge sources, including Isla Aguja (IA), Bahía Concha (BC), and Bahía Neguanje (NG); and (2) stations with the influence, by direct or diffuse discharges, including the submarine outfall (ES), Santa Marta Bay (BS), the Manzanares River (RM), and Playa Blanca (PB). The selection of these sampling sites was supported by previous studies (Moscarella, García & Palacio, 2011; Mancera, Pinto & Vilardy, 2013; García-Rentería, Nieto & Cortez, 2023; INVEMAR, 2024) (Table S1). As a reference for monthly precipitation, data from the Institute of Hydrology, Meteorology and Environmental Studies (IDEAM) were considered to define dry months (<100 mm) and rainy months (>100 mm)) (Table S2).

### Sample collection

**Figure 2:**
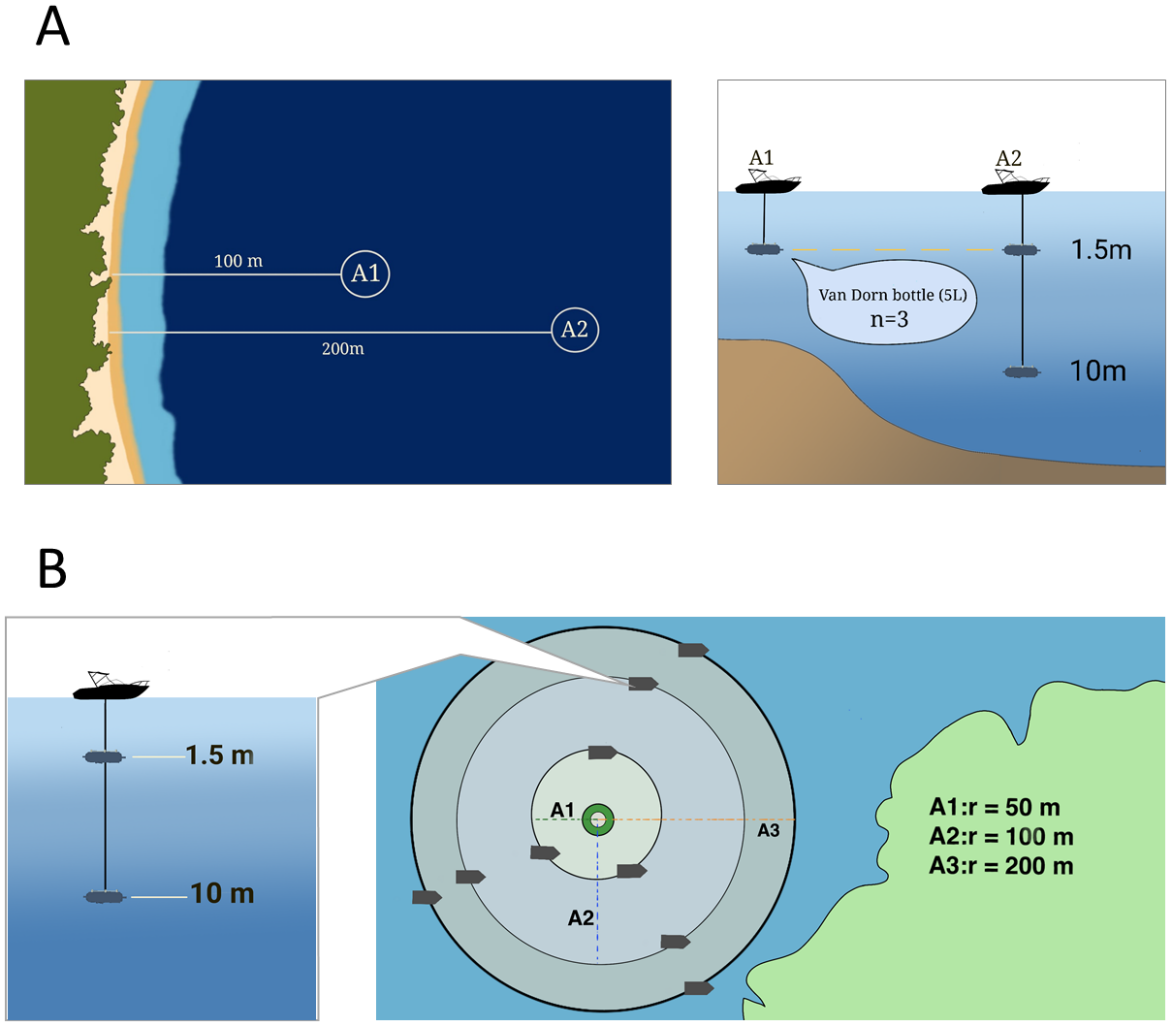
General scheme for coastal and marine sample collection at stations in the coastal zone of Santa Marta Bay. (A) Sampling at nearshore and offshore points at depths of 1.5 and 10 m. (B) Sampling conducted along three concentric rings (50, 100, and 200 m) dist.

Seawater samples were collected at each station during 14 field trips, from April 2021 to March 2024. Sampling occurred during: April, June, August, October, November, and December 2021; November and December 2022; February, May, July, September, and November 2023; and March 2024. At each station, two sampling points were designated: one nearshore and one offshore (Fig. 2A). An exception was made for the submarine outfall (ES), where three sampling points were arranged in three concentric rings around the discharge center at radii of 50, 100, and 200 m (Fig. 2B). At each field trip, four samples were collected at each station (totaling 4 L) using a Van Dorn bottle at depths of 1.5 m and 10 m (Cañón Páez et al., 2005). In cases where the total water column was less than 10 m, particularly at nearshore locations, samples were collected only at 1.5 m (Fig. 2B). Sampling was conducted between 08:30 and 11:45 a.m., beginning at the northernmost station and proceeding southward. A total of 432 seawater samples were collected. All samples were stored in pre-cleaned plastic bottles, maintained at 4 ^∘^C, and transported by boat (∼4 h) to the Water Quality Laboratory at the University of Magdalena for subsequent analysis.

### Physicochemical analysis

*In situ* measurements of temperature, salinity and conductivity were performed using an SI Analytics HandyLab 200 meter (accuracy ≤ 0.5% of the measured value ±1 digit). Dissolved oxygen (DO, mg L^−^^1^) was measured using a WTW Oxi 3310 meter (accuracy of ≤ 0.1 K ±1 digit), and pH was measured using a SI Analytics HandyLab 100 meter equipped with a WTW Sentix 41 pH electrode (accuracy of ≤ 0.005) (Table S3). The electrode was calibrated monthly using NBS buffers (pH 4.0, 7.0, and 10.0), and during each calibration, the slope was checked and consistently exceeded 97%, indicating a near-Nernstian response (Dickson, Sabine & Christian, 2007). Collected water samples were subsequently analyzed for three categories of variables: (1) Turbidity and oxygen demand parameters that included total suspended solids (TSS, mg L^−^^1^), turbidity (nephelometric turbidity units, NTU), biochemical oxygen demand (BOD_5_, mg L^−^^1^ O_2_), and chemical oxygen demand (COD, mg L^−^^1^ O_2_). (2) Pollutants, including fats and oils (mg L^−1^) and polycyclic aromatic hydrocarbons (PAHs) (µg L^−1^); and 3) Nutrients including nitrates (NO_3_^−^), nitrites (NO_2_^−^), phosphates (PO_4_^3−^) and ammonium (NH_4_^+^) (µg L^−1^). Water samples were filtered using 0.45 µm Whatman filter paper for nutrient analysis. When it was not possible to analyze them on the same day, the samples were frozen and analyzed within 72 h. The analytical procedures followed standard protocols by Rice, Baird & Eaton (2017) and Strickland & Parsons (1972), in accordance with Colombian environmental regulations for marine waters (Decree 1076 of 2015) (MinAmbiente, 2015), as well as the guidelines of the World Health Organization (World Health Organization, 2003) on water quality. Threshold values for recreational use and marine biota conservation were used as references for comparative assessment (Table S4).

### Estimation of the marine environmental quality index

Water quality was estimated by analyzing the averages and weighted values of the following variables: DO (mg L^−1^ O_2_), pH, TSS (mg L^−1^), BOD5 (mg L^−1^ O_2_), and nutrients (nitrates (NO_3_^−^), phosphates (PO_4_^3−^)) (µg L^−1^) and thermotolerant coliforms (NMP/100 ml) using factors “season” and “rainy-dry season”. Each variable was normalized and converted into a subindex contributing to the overall Marine Environmental Quality Index (ICAM). The ICAM was calculated using weighted averages through nonlinear equations that assign a score from 0 (poor quality) to 100 (excellent quality). Calculations were performed using the SiAM platform developed by INVEMAR, following the methodology of Vivas-Aguas (2011) and Castillo-Viana et al. (2022). The results were visualized in box plots depicting the distribution and central tendency of ICAM scores, categorized into five qualitative classes: optimal, adequate, acceptable, inadequate and very poor (Table S5).

### Interpolation analysis

The spatial characterization of physicochemical parameters was carried out across the different sampling stations. The results for each variable were grouped according to seasonal precipitation patterns, defining dry months as those with <100 mm of rainfall and rainy months as those with >100 mm, based on the Köppen-Geiger climate classification (Kottek et al., 2006). Average values of physicochemical parameters were calculated for each sampling station during the climatic seasons (dry and rainy) at depths of 1.5 m and 10 m. This approach was adopted to highlight distribution patterns and facilitate comparative analysis between the dry and rainy seasons. The resulting seasonal average values were visualized using gradient colors in spatial thematic maps created with QGIS software (*v.* 3.26.3).

### Statistical analysis

Data were treated prior analysis to avoid redundancy and co-linearity among variables. To assess multicollinearity, we performed a correlation analysis (Table S6) and visualized the relationships between variables using Draftsman plots, which indicated no redundancy among variables. Then, the data were log-transformed using the formula Log (x + 1), normalized, and used to construct a Euclidean distance matrix. This matrix was then employed for the main PERMANOVA analysis to assess differences among groups, with a 95% confidence level. The PERMANOVA model included the factors “season” and “climatic season-year”, with unrestricted permutations of residuals under a full model. Type III sums of squares were applied, assuming fixed effects summed to zero, and 999 permutations were executed. When significant differences were detected (*p* < 0.05), pairwise PERMANOVA comparisons and Monte Carlo *post hoc* tests were conducted. To identify the variables contributing most to observed dissimilarities among groups, a percentage similarity (SIMPER) analysis was performed. Multivariate patterns were visualized using principal component analysis (PCA) (Tables S7, S8 and S9). Fats/oils and PAH variables were excluded from both analysis due to the presence of missing values. All statistical analyses were conducted using PRIMER v7 (Clarke & Gorley, 2015), RStudio, and GraphPad Prism v10. Results were visualized using box plots and trend lines to illustrate means and variability (standard error) across categories.

## Results

### Temporal dynamics of physicochemical variables

Seawater temperature was below 26 °C during dry seasons and higher than 27 °C in rainy periods, peaking at 30.13 ± 0.79 °C in 2022. Salinity averaged 35.8 ± 0.15 in dry months but dropped to 31.86 ± 2.33 during the 2022 rainy season. The pH ranged from 8.04 ± 0.06 (dry) to 9.12 ± 0.36 (rainy 2022). Dissolved oxygen (DO) remained relatively uniform across the area, with values above 4.25 ± 0.20 mg O_2_ L^−1^ and reaching 5.78 ± 0.44 mg L^−1^ in the dry season of 2023. Oxygen saturation varied between 53.33 ± 3.78% and 76.91 ± 6.86% during the same period (Table 1).

**Table 1:** Mean values ± standard error (SE) of temperature, salinity, pH, dissolved oxygen (DO) and oxygen saturation measured along the coastal area of Santa Marta, Colombia, during dry and rainy months from 2021 to 2024.

	**Climatic period-year**						
	**Dry-2021**	**Rainy-2021**	**Dry-2022**	**Rainy-2022**	**Dry-2023**	**Rainy-2023**	**Dry-2024**
Temperature (°C)	26.29 ± 0.18	27.47 ± 0.44	26.21 ± 4.57	30.13 ± 0.84	25.85 ± 0.79	28.49 ± 1.42	26.14 ± 1.26
Salinity	35.82 ± 0.15	34.93 ± 0.59	35.39 ± 0.17	31.86 ± 2.33	34.81 ± 0.23	34.13 ± 1.02	34.65 ± 1.67
pH	8.04 ± 0.06	8.15 ± 0.07	8.15 ± 0.09	9.12 ± 0.36	8.38 ± 0.22	8.16 ± 0.63	8.21 ± 0.05
D.O. (mg O_2_ L^−1^)	4.25 ± 0.20	4.40 ± 0.72	5.48 ± 0.42	5.12 ± 0.66	5.78 ± 0.44	5.45 ± 0.81	4.31 ± 0.30
Saturation O_2_ (%)	53.88 ± 1.99	53.58 ± 3.70	73.00 ± 4.55	67.30 ± 6.92	76.91 ± 6.86	69.10 ± 11.63	53.33 ± 3.78

**Figure 3:**
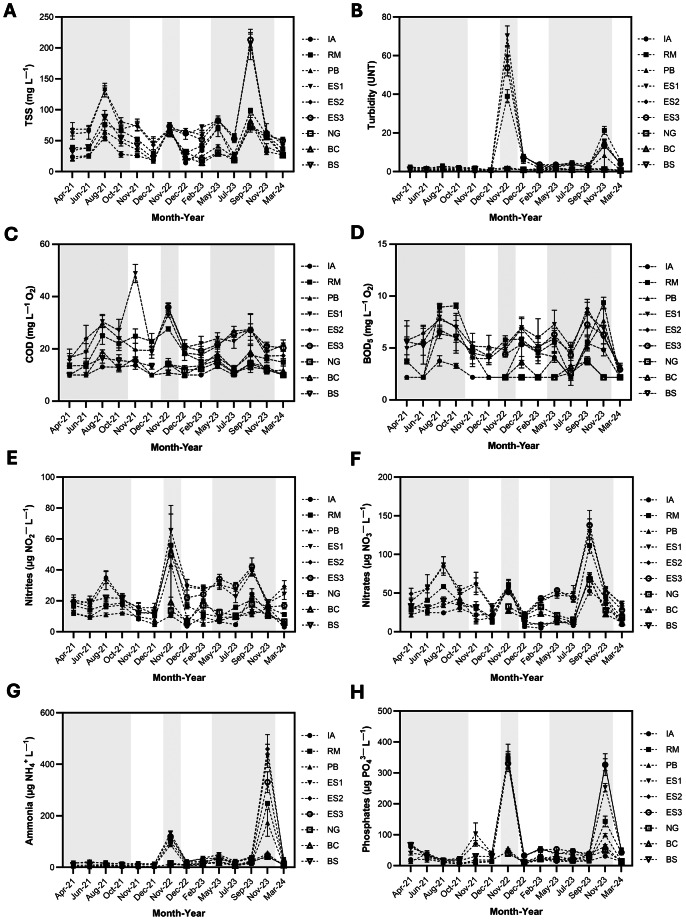
Spatial and temporal variation of physicochemical parameters over 14 months (2021–2024) and across sampling stations (IA, Isla Aguja; RM, Manzanares River; PB, Playa Blanca; ES1–ES3, Submarine outfall rings 1–3; NG, Neguanje; BC, Concha Bay; BS, Sa). Dashed lines indicate temporal trends; error bars represent mean ± SE. Dry months are highlighted in white, while rainy months are shaded in gray.

Elevated concentrations of total suspended solids (TSS) were detected during the rainy seasons of August 2021 (49–154.5 mg L^−1^), November 2022 (56.7–79.2 mg L^−1^), May 2023 (28.3–93.3 mg L^−1^) and September 2023 (67.5–235 mg L^−1^). These peaks were notably attenuated at stations within the protected area (IA, BC, NG) (Fig. 3A). Turbidity also increased markedly during the rainy periods, reaching 78.6 NTU in November 2022 and 23.4 NTU in November 2023, with the highest values recorded at the submarine outfall (ES1 and ES2), and RM (Fig. 3B).

BOD_5_ ranged from 2.2 to 9.5 mg L^−^^1^ O_2_ and increased significantly during August–October 2021 and September–November 2023, corresponding with peak rainfall periods (Table S2, Fig. 3C). Stations ES, BS, RM, and PB consistently exceeded the regulatory threshold of 5 mg L^−^^1^ O_2_. Similarly, COD varied between 10 and 34.5 mg L^−^^1^ O_2_, with the highest concentrations observed in August–November 2021, November 2022, and September 2023. Maximum COD values (34.5 mg L^−^^1^ O_2_) were recorded at ES1, ES2, and BS in November 2021 (Fig. 3D), whereas other stations remained below the regulatory limit of 30 mg L^−^^1^ O_2_ (Table S4). The COD/BOD_5_ ratio in coastal seawater from Santa Marta showed higher values during the rainy season, ranging from 4.48 to 8.20 at ES, 2.52 to 6.25 at RM, and 2.59 to 3.33 at BS. In contrast, lower ranges were recorded at the PB, IA, BC, and NG stations (Table S10).

Nutrients concentrations exhibited spatiotemporal patterns similar to those of BOD_5_ and COD (Figs. 3E–3H). Nitrate levels ranged from 4.0 and 167.6 µg NO_3_^−^ L^−1^, with pronounced increases in August and November 2021, and November 2022, particularly at the ES substations (ES1–ES3) and RM. A widespread increase across all stations was observed in September 2023, with peak concentrations (>115 µg NO_3_^−^ L^−1^) recorded at RM and the outfall substations (Fig. 3E). Nitrite concentrations (3.6–83 µg NO_2_^−^ L^−1^) exceeded 35 µg NO_2_^−^ L^−1^ during August 2021, November 2022 and May–September 2023, with the highest value occurring at RM and ES (Fig. 3F). Ammonia remained low for most of the months, except in November 2022 and especially in November 2023, when values spiked to 233–523 µg NH_4_^+^ L^−1^ at the outfall substations, RM, and PB (Fig. 3G). Phosphate levels were high in November 2022 and November 2023, reaching 305–404.7 µg PO_4_^3+^ L^−1^ at stations along Santa Marta Bay, and 33.7–53.6 µg PO_4_^3+^ L^−1^ within the TNNP (Fig. 3H). Exceedances of regulatory thresholds were primarily observed for nitrites, phosphates, and ammonium at the ES, RM, and PB stations during the rainy seasons of 2022 and 2023. Phosphate levels at the outfall substations, as well as at RM, PB, and BS, consistently exceeded the regulatory limit of 34 µg PO_4_^3+^ L^−1^ during these periods.

**Figure 4:**
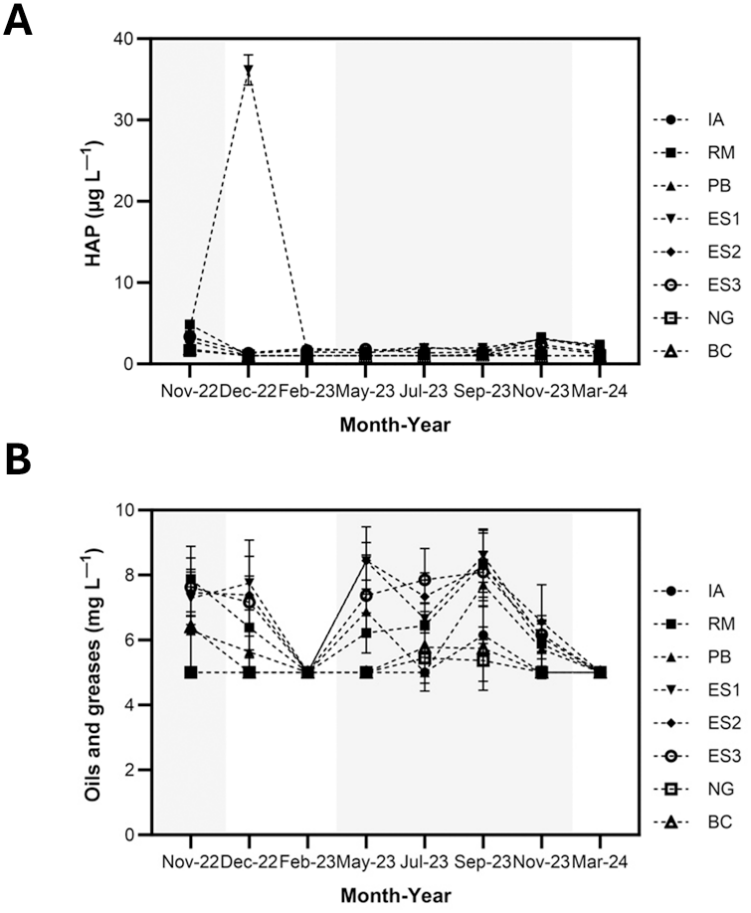
Spatial and temporal variation of physicochemical parameters over 14 months (2021–2024) and across sampling stations (IA, Isla Aguja; RM, Manzanares River; PB, Playa Blanca; ES1–ES3, Submarine outfall rings 1–3; NG, Neguanje; BC, Concha Bay; BS, Sa. Dashed lines indicate temporal trends; error bars represent mean ± SE. Dry months are highlighted in white, while rainy months are shaded in gray.

Fats and oils were detected in both dry (2023–2024) and rainy (2022–2023) seasons, with concentrations consistently exceeding the regulatory threshold for seawater (5 mg L^−1^). Elevated levels were observed at the RM and ES stations (>8 mg L^−1^), whereas significantly lower concentrations (1 mg L^−1^) were obtained at IA (Isla Aguja) station (Fig. 4A). Polycyclic aromatic hydrocarbons (PAHs) reached the highest concentrations (3.3–5.3 µg L^−1^) during the rainy periods of November 2022 and 2023, particularly at ES and RM. Notably, a maximum value of 36 µg L^−1^ was recorded at ES1 in December 2022, substantially exceeding the permissible limit of 0.05 µg L^−1^ (Fig. 4B).

All evaluated physicochemical variables exhibited distinct spatial patterns influenced by the proximity and intensity of wastewater discharge, as well as temporal variability associated with climatic seasonality (dry *vs.* rainy periods). Significant differences were observed over the years between sampling stations (PERMANOVA; pseudo-F(8, 424) = 43.03, *p* = 0.001, p-adjusted = 0.001), across climatic seasons (PERMANOVA; pseudo-F(6, 426) = 46.41, *p* = 0.001, p-adjusted = 0.001), and for combined spatiotemporal interactions (PERMANOVA; pseudo-F(37, 395) = 2.42, *p* = 0.001, p-adjusted = 0.001). Pairwise comparisons showed significant seasonal differences across most sampling periods (*p* < 0.05), except during the rainy season of 2023, when stations IA, PB, NG, and BC did not differ significantly (*p* > 0.05). In contrast, ES consistently differed from all other stations across all sampling periods (Table 2). SIMPER analysis identified key variables driving temporal differences: BOD5 (22.43%) during the rainy season of 2021, COD (30.56%) in the dry season of 2021, turbidity (35.86%) during the rainy season of 2022, TSS during both the dry (19.41%) and rainy (15.83%) season of 2023, and nitrites during the dry season of 2024 (Tables S7, and S8). Spatial variation was predominantly influenced by phosphates, nitrates, ammonium and BOD5 in Santa Marta Bay (∼16% contribution), while within TNNP, nitrites, nitrates, and TSS accounted for the greatest variability (14.23–32.74%) (Tables S7 and S8).

**Table 2:** Results of paired PERMANOVA between sampling stations based on dry and rainy months. ES, submarine outfall; IA, Isla Aguja; PB, Playa Blanca; RM, Manzanares River; NG, Neguanje Bay; BC, Concha Bay; BS, Santa Marta Bay.

	**Month-year**						
**Stations**	**Dry-2021**	**Rainy-2021**	**Dry-2022**	**Rainy-2022**	**Dry-2023**	**Rainy-2023**	**Dry-2024**
ES-IA	0.001^**^	0.001^**^	0.001^**^	0.001^**^	0.001^**^	0.001^**^	0.009^**^
ES-PB	0.001^**^	0.001^**^	0.015^**^	0.001^**^	0.001^**^	0.001^**^	0.017^**^
ES-RM	0.001^**^	0.001^**^	0.020^**^	0.005^**^	0.001^**^	0.001^**^	0.020^**^
ES-NG	–	–	0.015^**^	0.001^**^	0.001^**^	0.001^**^	0.012^**^
ES-BC	–	–	0.014^**^	0.001^**^	0.001^**^	0.001^**^	0.018^**^
ES-BS	0.001^**^	0.001^**^	–	–	–	–	–
IA-PB	0.119	0.069	0.001^**^	0.001^**^	0.001^**^	0.446	0.001^**^
IA-RM	0.003^**^	0.001^**^	0.001^**^	0.001^**^	0.001^**^	0.027^**^	0.001^**^
IA-NG	–	–	0.001^**^	0.001^**^	0.001^**^	0.175	0.001^**^
IA-BC	–	–	0.001^**^	0.001^**^	0.001^**^	0.123	0.001^**^
IA-BS	0.002^**^	0.001^**^	–	–	–	–	–
PB-RM	0.001	0.023^**^	0.001^**^	0.001^**^	0.001^**^	0.340	0.001^**^
PB-NG	–	–	0.001^**^	0.001^**^	0.001^**^	0.094	0.001^**^
PB-BC	–	–	0.001^**^	0.001^**^	0.001^**^	0.246	0.001^**^
PB-BS	0.001^**^	0.010^**^	–	–	–	–	–
RM-NG	–	–	0.001^**^	0.001^**^	0.001^**^	0.007^**^	0.001^**^
RM-BC	–	–	0.001^**^	0.001^**^	0.001^**^	0.039^**^	0.001^**^
RM-BS	0.084	0.048^**^	–	–	–	–	–
NG-BC	–	–	0.001^**^	0.001^**^	0.001^**^	0.500	0.094

**Notes.**

** Represents significant differences (*p* < 0.05).

**Figure 5:**
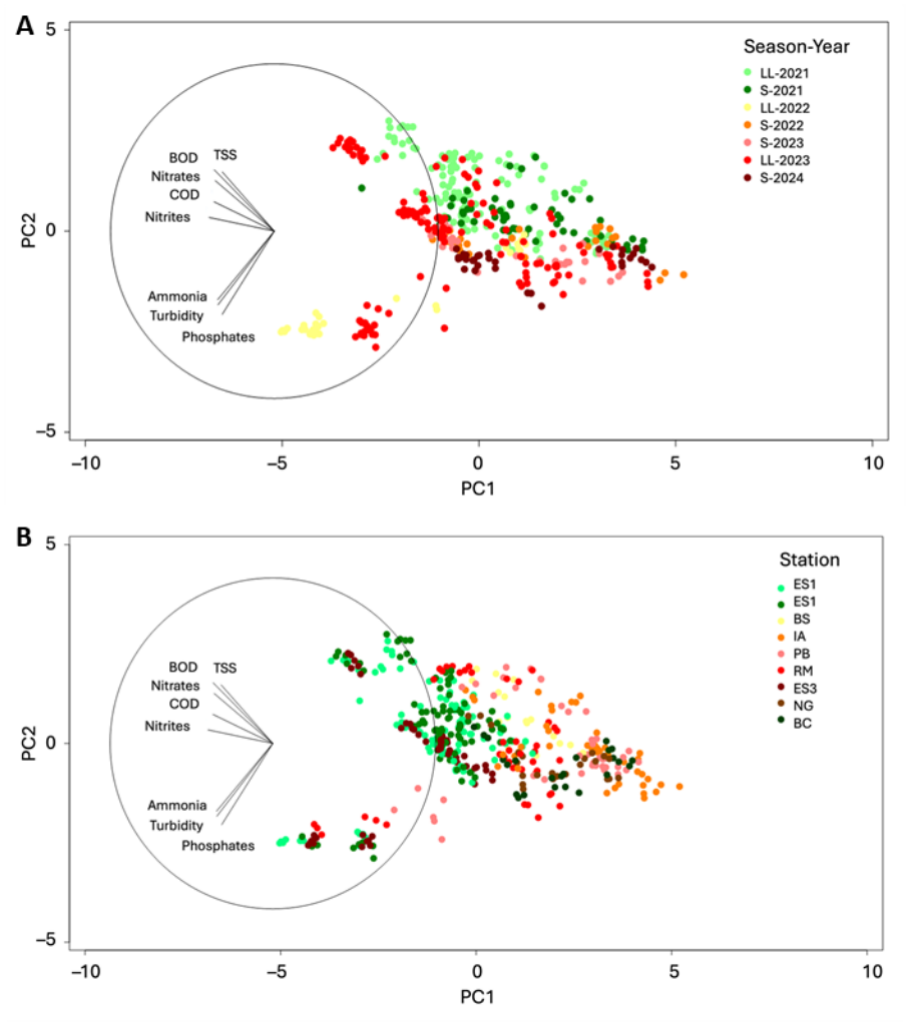
Principal Component Analysis (PCA). (A) Variation throughout the dry and rainy months from 2021 to 2024. (B) Variation across sampling stations (IA, Isla Aguja; RM, Manzanares River; PB, Playa Blanca; ES1–ES3, Submarine outfall rings 1–3; NG, Neguanje.

PCA showed that nitrates, TSS, BOD5, COD and nitrites were the primary drivers of spatiotemporal variability during the rainy seasons of 2021 and 2023, particularly at outfall-adjacent stations ES1, ES2, ES3, RM, and PB (Fig. 5B). Phosphates, ammonia, and turbidity contributed most to the variability observed during the rainy season of 2022 and were strongly associated with the ES and BS stations in Santa Marta Bay (Fig. 5A). Notably, these variables accounted for less variation during the rainy seasons of 2022 and 2023, and at stations IA, NG, BC, PB, and RM (Table S1). This suggests increased spatial homogeneity across Santa Marta Bay during these periods, regardless of site classification (impacted *vs.* protected) or sampling location (onshore *vs.* offshore) (Fig. 5B).

### Spatial distribution of physicochemical parameters

**Figure 6:**
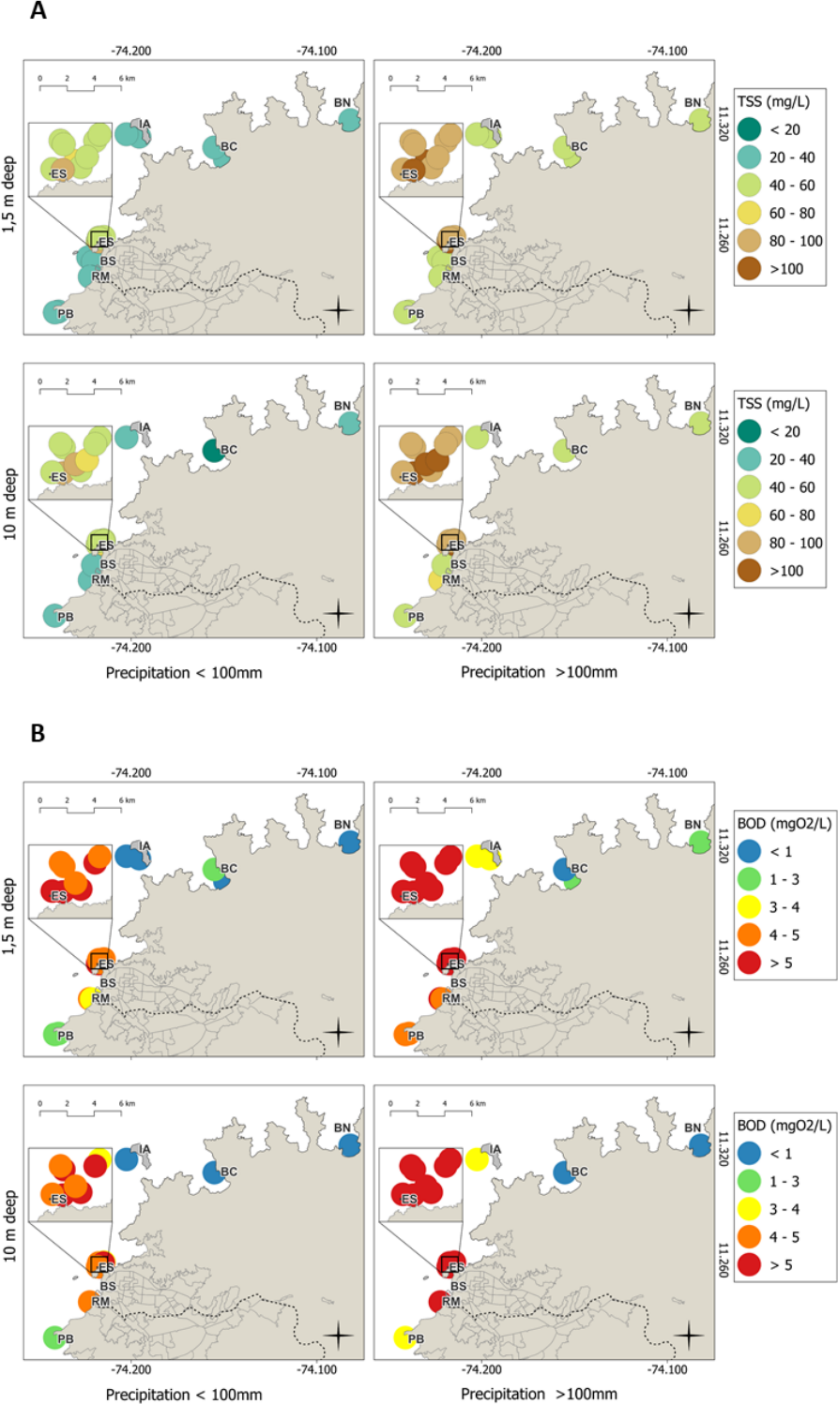
Spatial distributions of (A) total suspended solids (TSS) and (B) biological oxygen demand (BOD_5_) throughout the sampling stations in the coastal area of Santa Marta; results based on precipitation during the dry months (<100 mm) and rainy months.

**Figure 7:**
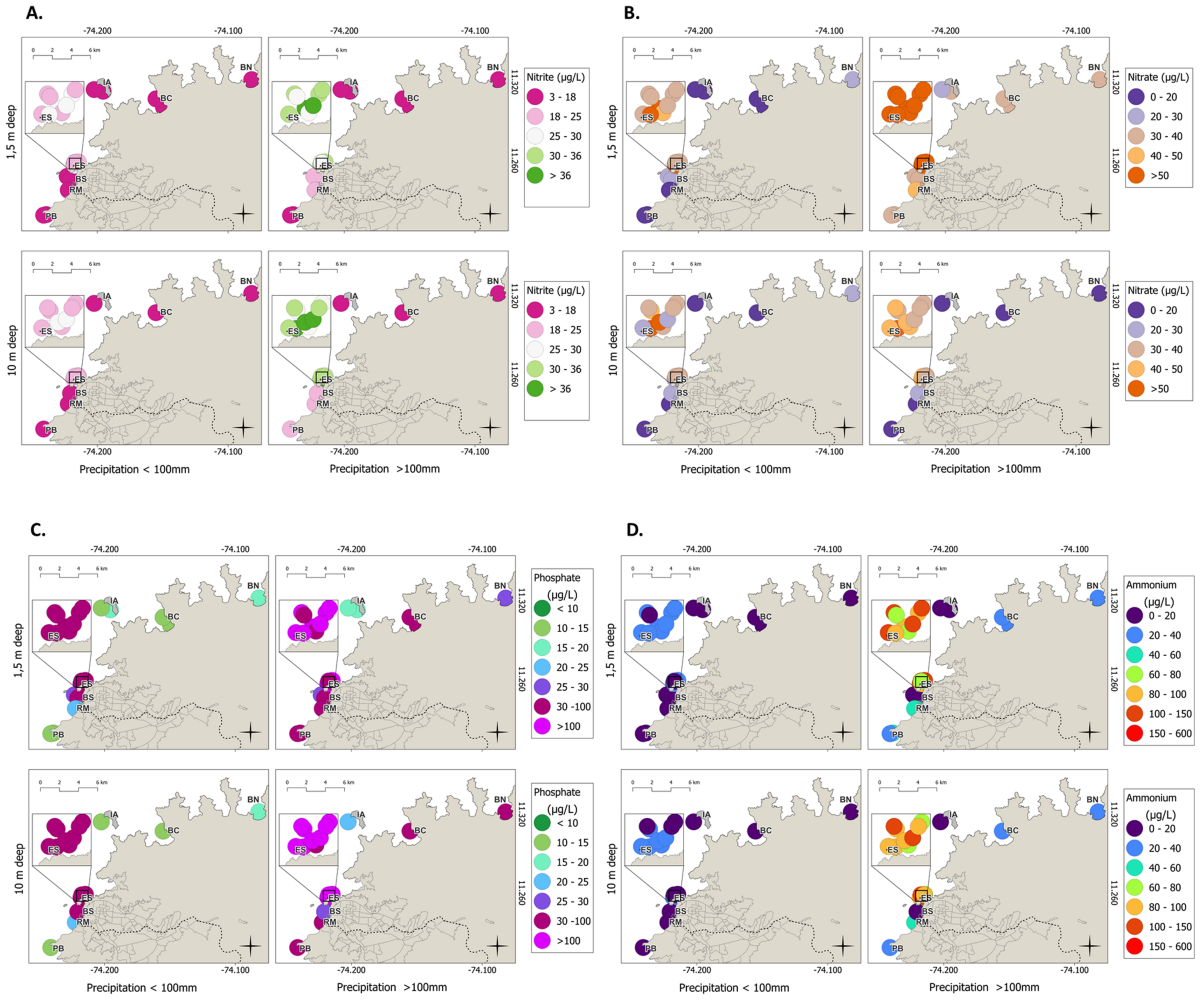
Spatial distribution across sampling stations in the coastal area of Santa Marta; results based on precipitation during dry months (<100 mm) and rainy months (>100 mm); and spatial distribution at depths of 1.5 and 10 m.

TSS, BOD_5_ (Figs. 6A–6B), and dissolved inorganic nutrients (ammonium, nitrites, nitrates, and phosphates; Figs. 7A–7D) exhibited clear spatial and temporal patterns modulated by rainfall. During dry periods (rainfall <100 mm), pollutant distributions were relatively uniform across depths (1.5 and 10 m), suggesting a stable, stratified water column with minimal influence from terrestrial runoff. Conversely, during the rainy seasons (rainfall >100 mm), concentrations became more heterogeneous, particularly at the surface (1.5 m) near discharge points. This pattern is consistent with increased vertical mixing and enhanced input from continental and urban runoff. Notably, the highest concentrations of BOD_5_, TSS, and nutrients at 10 m depth were recorded at ES, BS, and RM stations subject to intense anthropogenic pressure, wastewater discharge, and port-related activity. These hotspots may serve as sources of contamination that disperse toward adjacent stations such as IA and PB, suggesting a gradient of impact radiating from urban and industrial sources toward more protected areas (Figs. 6–7).

### Marine environmental quality index

**Figure 8:**
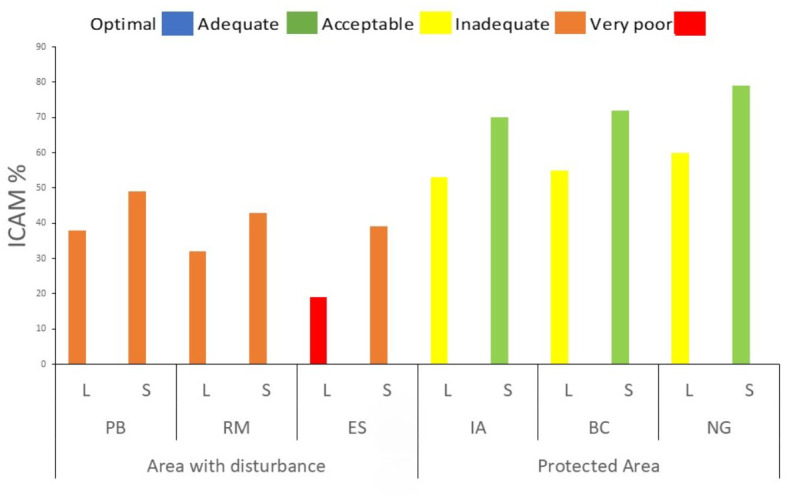
Spatial and temporal distributions of the Marine Environmental Quality Index (ICAM) across sampling stations in the coastal area of Santa Marta and results based on precipitation during dry (S) and rainy (L) months.

The ICAM generally was deteriorated during the rainy season (precipitation > 100 mm) across all stations. Overall, water quality ranged from inadequate (<50%) to adequate (ICAM > 50%), except at ES, which consistently showed “very poor” conditions in both the dry and rainy seasons (39% ± 12.73 and 19% ± 11.29, respectively). RM showed ICAM values of 32% ± 23.21 during the rainy season and 43% ± 6.56 during the dry season, indicating a moderate improvement due to the reduced influence of runoff. These low values demonstrate significant environmental degradation, likely caused by river inflows from station RM and direct wastewater discharges at ES (Fig. 8). Stations within the TNNP: IA, BC, and NG maintained acceptable water quality (ICAM > 50%) even during periods of increased precipitation, indicating a buffering effect of the protected area. Notably, the PB station showed a marked improvement during the dry season, with ICAM increasing from ∼38% ± 23.7 to ∼49% ± 7.57, though it remained within the inadequate quality range (Fig. 8, Table S11).

## Discussion

The analysis of spatiotemporal patterns of marine water quality in the Santa Marta region reveals climatic and anthropogenic influences and provides a novel approach by integrating the dynamics of precipitation, water discharge, and residual discharge with the environmental response of specific stations. While relative homogeneity was maintained in certain areas, stations such as ES and RM demonstrated marked declines in water quality, signalling localized environmental stress. These impacts, were exacerbated during the rainy seasons, highlights the critical role of runoff, the submarine outfall, and fluvial inputs from the Manzanares River.

### Temporal variation

The results show a clear increase in turbidity, COD, PAHs, nitrites, ammonia, phosphates and temperature during the rainy season compared to the dry period. The temperature increase is due to stratification of the water column, and a thermocline up to 12 m thick can be present (Arroyave Gómez et al., 2020; García-Rentería, Nieto & Cortez, 2023). Notably, 2022 exhibited the highest concentrations across all parameters and stations. This value is mainly attributed to the exceptionally high rainfall recorded (301.81 mm), which intensified continental runoff and increased the input of nutrients and organic matter into the coastal system. Additionally, greater pressure from wastewater discharges during this period further disrupted biogeochemical cycles, enhancing eutrophication processes and amplifying impacts on ecosystem functioning and biodiversity (The MerMex Group et al., 2011; Kunzmann et al., 2022; García-Rentería, Nieto & Cortez, 2023; Xiao et al., 2024; Sanchez-Barranco et al., 2025; Dehm et al., 2025).

Elevated concentrations of fats and oils observed during the rainy seasons, particularly at ES station are likely attributable to the discharge of untreated domestic wastewater and urban runoff. These pollutants, commonly originating from residential, industrial, and commercial sources, and are transported *via* stormwater and sewer systems to the submarine outfall, which releases effluents directly into the coastal zone. Similar patterns of accumulation near outfalls, port areas, and river mouths during periods of high precipitation have been reported by Roveri et al. (2021) and Domingo & Manejar (2021). Fats and oils in wastewater can constitute up to 10% of the total pollutant load, with typical concentrations ranging from 10 to 100 mg L^−^^1^ (Eljaiek-Urzola et al., 2019). Their presence in coastal waters significantly compromises water quality and affects sensitive ecosystems such as mangroves and soft-bottom habitats. These substances form persistent surface films that reduce light penetration, impair photosynthetic activity, and deplete dissolved oxygen, leading to asphyxia in aquatic organisms. Furthermore, degradation of lipid compounds can generate toxic byproducts capable of inducing fish mortality and ecosystem imbalances (Hussein et al., 2022; Francis, Estim & Sidik, 2024). The exceptionally low values recorded in February 2023 and March 2024 (<5 mg L^−1^) may be attributed to the decrease in precipitation (4.37 mm in February 2023 and 45.19 mm in March 2024) compared to the other sampling periods. Likewise, the presence of strong trade winds and coastal upwelling during this period, particularly between December and March (Arroyave Gómez et al., 2020), may have contributed to this situation. These processes likely promote greater dilution of contaminants in the water column, in addition to the intense surface waves typical of the dry season, which also facilitate their dispersion (García-Rentería, Nieto & Cortez, 2023).

Oily wastewater is also a carrier of hazardous compounds such as phenols and polycyclic aromatic hydrocarbons (PAHs), which are known to be mutagenic, carcinogenic, and growth-inhibitory to aquatic life (Soares et al., 2021). These findings underscore the critical need for improved wastewater treatment and runoff management to mitigate pollutant loads in coastal environments, particularly during rainfall-driven discharge events. This study also revealed that PAHs exceeded permissible concentrations more frequently during the rainy season, indicating an early warning for contamination in the coastal waters of Santa Marta Bay. The primary source appears to be the submarine outfall. Although the direct impacts of PAHs on local marine biota remain insufficiently characterized, their environmental persistence, toxicity, and capacity for bioaccumulation in food webs warrant the establishment of precautionary thresholds (Cheng et al., 2023; Hussein et al., 2022).

Elevated nutrient levels including nitrates, nitrites, ammonium, and phosphates, have previously been documented in Santa Marta Bay (García, Palacio & García, 2012; Bayraktarov, Bastidas-Salamanca & Wild, 2014). The proximity to the submarine outfall, which releases virtually untreated effluents containing high organic and nutrient loads, is a likely driver of this pollution (Arroyave Gómez et al., 2020). These discharges, at depths of 22–28 m and distances of ∼1.8 km from the coast, contribute to the lateral transport of nutrients and pollutants toward ecologically sensitive sites such as IA and TNNP, which host important coral reefs, seagrass beds, and recreational areas (Vega-Sequeda et al., 2008; Ruiz-Toquica et al., 2023). Nutrient enrichment in tropical coastal systems is known to drive macroalgal blooms, reduce coral cover, and disrupt benthic community structure (Wiedenmann et al., 2013; Gómez-Cubillos et al., 2019; Gómez-Cubillos, Gavio & Zea, 2020). Excessive nutrient loads can also alter microbial communities by suppressing biomass, increasing diversity, and disrupting ecological balance (Tao et al., 2017). These impacts can be assessed through bioindicators in planktonic and benthic assemblages within mangrove ecosystems (Alonso et al., 2009; García-Rentería, Nieto & Cortez, 2023).

The COD/BOD_5_ ratio between the urban stations and the more conserved areas reveals marked contrasts in the quality of organic matter in the coastal waters of Santa Marta. At the impacted stations (ES, RM, and BS), the highest ratios reflecting the presence of refractory compounds associated with wastewater discharges and urban runoff. This pattern is consistent with studies indicating that values above 2.5 are characteristic of environmentally stressed systems receiving inputs of poorly biodegradable organic matter (Metcalf, Eddy & Tchobanoglous, 2004; Wu et al., 2021; Kashkouli, Partovinia & Ghorbannezhad, 2025). Such conditions can reduce oxygen availability and alter microbial and biogeochemical processes within the system (Wu et al., 2021). In contrast, the stations located in areas with lower anthropogenic influence (IA, BC, and NG) exhibited lower COD/BOD_5_ ratios, suggesting a higher proportion of biodegradable organic matter and a more stable ecological functioning. This pattern aligns with observations from other tropical environments, where conserved areas maintain more balanced cycles of organic matter input and degradation (Jiang et al., 2022). These differences between disturbed and conserved areas are further supported by the behavior of other environmental indicators, particularly suspended solids (TSS), which also reveal strong spatial and seasonal gradients in response to human impact and hydrometeorological conditions.

The pronounced TSS peaks observed during rainy periods in 2021–2023 (ranging from 28.3 to 235 mg L^−^^1^) reflect enhanced sediment mobilization driven by continental runoff and discharges from the ES and the RM, while stations within the protected area consistently showed much lower TSS levels. This pattern aligns with Fong, Gaynus & Carpenter (2020), who reported that suspended solids are largely mobilized by precipitation driven runoff. In Santa Marta Bay, these solids are composed mainly of sand and fine sediments are transported *via* stormwater and riverine flows (Moscarella, García & Palacio, 2011) and act as vectors for pathogenic bacteria that adversely affect benthic fauna, particularly corals (Witt, Wild & Uthicke, 2012; Rogers & Ramos-Scharrón, 2022). Although the impacts of sedimentation on coral reef degradation have been documented locally (Bayraktarov, Bastidas-Salamanca & Wild, 2014; Gómez-Cubillos et al., 2019; Ruiz-Toquica et al., 2023), the specific sources and characteristics of sediment contamination remain insufficiently described. The persistence of suspended solids associated with both anthropogenic discharges and natural runoff underscores the need for a more detailed assessment of their origin, transport pathways, and ecological implications, especially given the sensitivity of coral reef ecosystems.

### Spatial variation

The spatiotemporal analysis of water quality revealed clear patterns driven by both climatic variability and anthropogenic pressures. While some sites exhibited relative stability regardless of continental discharge influence, stations ES and RM showed a decline in water quality, indicating localized environmental stress. These effects were particularly evident during the rainy season, when precipitation, surface runoff, wastewater discharges, and fluvial inputs, especially from the Manzanares River and the submarine outfall system-intensified the degradation of water conditions.

Likewise, the spatiotemporal patterns of physicochemical variables (TSS, BOD_5_, and nutrients such as ammonium, nitrites, nitrates, and phosphates) confirmed the influence of rainfall periods and anthropogenic activity in Santa Marta Bay and TNNP. During the dry months, the distribution of these parameters was relatively homogeneous between 1.5 m and 10 m depths, reflecting stable water columns with minimal terrestrial influence. In contrast, rainy periods were characterized by elevated TSS and nutrient concentrations. Similar seasonal dynamics have been reported along the Quintana Roo coast, Mexico, where dry seasons show low turbidity and nutrient levels, while wet seasons exhibit significant enrichment due to intensified terrestrial discharges (Hernández-Terrones et al., 2015). Comparable findings have been documented in Cartagena Bay, Colombia, where increases in TSS and nutrients are linked to rainfall, urban discharges, and riparian deforestation, processes that collectively enhance the transport of sediments and contaminants to marine systems (Restrepo-López et al., 2015).

During rainy months, surface concentrations (1.5 m) of TSS and BOD_5_ showed increased variability near discharge sites due to enhanced vertical mixing and continental inputs of organic matter (Smith, Joye & Howarth, 2006; Torregroza-Espinosa et al., 2020). Notably, BOD_5_, nutrients, and TSS peaked at 10 m depth at ES, BS, and RM stations with intense anthropogenic pressure and residual discharges. These stations appear to function as sources of pollution, with potential downstream impacts on PB, IA, BC, and NG, forming a gradient of eutrophication and declining water quality from impacted zones to relatively undisturbed areas (García, Palacio & García, 2012). This eutrophication gradient aligns with findings from García-Urueña, Garzón-Machado & Zárate-Arévalo (2025) at Isla Aguja, where *Acropora palmata* populations suffer from ecological degradation linked to poor water quality, increased pollutants, and human proximity. Elevated nitrate and phosphate concentrations are known to drive eutrophication, macroalgal proliferation, and coral decline, especially during rainy seasons, which enhance organic matter (Lapointe, Barile & Matzie, 2004; Fabricius, 2005; Carriquiry et al., 2001; Guzman, Cipriani & Jackson, 2008).

### Marine environmental quality index

Stations near ES, RM, and BS consistently exhibited inadequate to poor water quality, conditions that not only restrict recreational activities but also signal chronic nutrient and organic matter loading from wastewater inputs and riverine discharges. These patterns reflect the cumulative impacts of urban expansion, insufficient sewage treatment, and fluctuating hydrological regimes, which together drive eutrophication and reduce environmental resilience. In contrast, stations IA, BC, and NG maintained acceptable conditions that support marine life and recreational use, illustrating the buffering capacity of well-managed protected areas. Comparable trends have been documented in other Caribbean MPAs such as Bonaire and Belize, where regulated zones maintain superior water quality relative to unprotected coastal areas (Roberts & Hawkins, 2000). Recent assessments by INVEMAR also report optimal conditions within TNNP (INVEMAR, 2024; Vivas-Aguas et al., 2024), reinforcing the pivotal role of MPAs in preserving coral reef health and broader marine biodiversity (Ruiz-Toquica et al., 2023). These findings lead us to propose improving wastewater treatment infrastructure in the Bay of Santa Marta and implementing stricter regulatory measures on riverine inputs, as well as the possible expansion of protected areas to strengthen environmental management in the region. Such actions are essential to protect public health, mitigate environmental degradation, and preserve marine ecosystem services.

## Conclusions

The spatiotemporal analysis of the physicochemical dynamics of seawater in the Bay of Santa Marta and TNNP between 2021 and 2024 revealed significant seasonal and anthropogenic influences on water quality. During the rainy periods, higher concentrations of nutrients, organic pollutants, turbidity, and oxygen-demanding compounds, including solids, were observed, particularly at stations impacted by wastewater discharges such as ES and RM. These pollutant levels frequently exceeded the maximum permissible limits, leading to water quality degradation and negatively affecting ecosystem stability especially seagrass beds and coral reefs as well as public health. A clear eutrophication gradient was identified, extending from heavily impacted stations (ES, BS, RM) toward more pristine sites (IA, BC, NG, PB) exhibiting better seawater quality.

The ICAM index confirming poor water quality near sewage, riverine and port discharge points, while stations within TNNP maintained acceptable environmental conditions. These findings highlight the beneficial role of MPAs in preserving water quality and emphasize the urgent need to modernize wastewater treatment infrastructure and enforce effective controls on urban and riverine discharges. Moreover, it is recommended to establish more targeted and periodic monitoring that integrates *in situ* sensors and intensive sampling campaigns during the rainy seasons, as well as to expand the network of stations within protected zones to enhance spatial resolution and enable early detection of potential water quality alterations. These actions will strengthen adaptive management and the sustainable conservation of coastal ecosystems in the Colombian Caribbean.

##  Supplemental Information

10.7717/peerj.20855/supp-1 Supplemental Information 1 Sampling site locations sites with minor or no influence from water discharges: NG (Neguanje), BC (Concha Bay), IA (Isla Aguja); Sites highly influenced by water discharges: PB (Playa Blanca), ES (Submarine Outfall), BS (Santa Marta Bay), and RM (Manzanares River). Sampling points A1, A2 and correspond to Ring 1, Ring 2 and Ring 3, EAF refers to offshore points, EC to coastal points. Coordinates follow the MAGNA-SIRGAS/Colombia Bogotá Zone format (EPSG 3116).

10.7717/peerj.20855/supp-2 Supplemental Information 2 Classification of sampling months based on average monthly rainfall (mm), using data from IDEAM Months with rainfall <100 mm were categorized as dry, while those with rainfall <100 mm were classified as rainy.

10.7717/peerj.20855/supp-3 Supplemental Information 3 Equipment, Technical Specifications, and Standards for Nutrient Analysis

10.7717/peerj.20855/supp-4 Supplemental Information 4 Physicochemical variables assessed. The table shows the regulated maximum seawater quality values for recreational use and for the conservation of marine biota, as well as the analytical detection method used The regulated maximum seawater quality values for recreational use and for the conservation of marine biota, as well as the analytical detection method used.
NTU = nephelometric units of turbidity.

10.7717/peerj.20855/supp-5 Supplemental Information 5 Assessment Scale for the Marine Environmental Quality Index (ICAM)

10.7717/peerj.20855/supp-6 Supplemental Information 6 Correlation analysis between physicochemical variables Correlations suggest no redundancy in between variables.

10.7717/peerj.20855/supp-7 Supplemental Information 7 SIMPER analysis during the dry and rainy season 2021-2022

10.7717/peerj.20855/supp-8 Supplemental Information 8 SIMPER analysis during the dry and rainy season 2022-2024 and 2024

10.7717/peerj.20855/supp-9 Supplemental Information 9 Eigenvalues of PCA

10.7717/peerj.20855/supp-10 Supplemental Information 10 COD/BOD ratio at sampling sites

10.7717/peerj.20855/supp-11 Supplemental Information 11 Calculation of the marine environmental quality index (ICAM) at sampling stations during dry and rainy periods

10.7717/peerj.20855/supp-12 Supplemental Information 12 Physicochemical parameters statistics

10.7717/peerj.20855/supp-13 Supplemental Information 13 ICAM Database 2

## Additional Information and Declarations

### Data Availability

The following information was supplied regarding data availability: The raw data are available in the Supplementary Files.
