# Supplementary material for: Physicochemical water quality in coastal marine ecosystems: spatiotemporal variation between protected and disturbed areas
Source: PeerJ. 2026 Mar 19;14:e20855. doi: 10.7717/peerj.20855 (PMC13006004; doi:10.7717/peerj.20855)
Supplement: Supplemental Information 1 — sites with minor or no influence from water discharges: NG (Neguanje), BC (Concha Bay), IA (Isla Aguja); Sites highly influenced by water discharges: PB (Playa Blanca), ES (Submarine Outfall), BS (Santa Marta Bay), and RM (Manzanares River). Sampling points A1, A2 and correspond to Ring 1, Ring 2 and Ring 3, EAF refers to offshore points, EC to coastal points. Coordinates follow the MAGNA-SIRGAS/Colombia Bogotá Zone format (EPSG 3116). [file peerj-14-20855-s001.docx]

| **Location** | **Station** | **Sampling point** | **Sampling depth** | **Distance from the coast** | **N Coordinates** | **W Coordinates** |
| --- | --- | --- | --- | --- | --- | --- |
| Less or not influenced by water discharges | NG | EAF | 1.5 y 10 m | 200 m | 11,352548 | –74,086050 |
|  |  | EC | 1.5 m | 50 m | 11,351400 | –74,084900 |
|  | BC | EAF | 1.5 y 10 m | 200 m | 11,300853 | –74,164194 |
|  |  | EC | 1.5 m | 50 m | 11,299100 | –74,053100 |
|  | IA | EAF | 1.5 y 10 m | 200 m | 11,31338 | –74,19472 |
|  |  | EC | 1.5 m | 50 m | 11,31422 | –74,19524 |
| Highly influenced by water discharges. | ES | A1-01 | 1.5 y 10 m | 50 m | 11,25893 | –74,21601 |
|  |  | A1-02 | 1.5 y 10 m | 100 m | 11,25763 | –74,21965 |
|  |  | A1-03 | 1.5 y 10 m | 200 m | 11,25630 | –74,22000 |
|  |  | A2-01 | 1.5 y 10 m | 50 m | 11,25632 | –74,21996 |
|  |  | A2-02 | 1.5 y 10 m | 100 m | 11,25618 | –74,21680 |
|  |  | A2-03 | 1.5 y 10 m | 200 m | 11,25695 | –74,21571 |
|  |  | A3-01 | 1.5 y 10 m | 50 m | 11,25817 | –74,21639 |
|  |  | A3-02 | 1.5 y 10 m | 100 m | 11,25841 | –74,21857 |
|  |  | A3-03 | 1.5 y 10 m | 200 m | 11,25736 | -74,21510 |
|  | BS | EAF | 1.5 y 10 m | 200 m | 11,24817 | –74,22034 |
|  |  | EC | 1.5 m | 50 m | 11,24596 | –74,21603 |
|  | RM | EAF | 1.5 y 10 m | 200 m | 11,23750 | –74,22331 |
|  |  | EC | 1.5 m | 50 m | 11,24819 | –74,22034 |
|  | PB | EAF | 1.5 y 10 m | 200 m | 11,21785 | –74,24203 |
|  |  | EC | 1.5 m | 50 m | 11,21935 | –74,23983 |

**Supplementary Table 1.** Sampling site locations. Sites with minor or no influence from water discharges: NG (Neguanje), BC (Concha Bay), IA (Isla Aguja); Sites highly influenced by water discharges: PB (Playa Blanca), ES (Submarine Outfall), BS (Santa Marta Bay), and RM (Manzanares River). Sampling points A1, A2 and correspond to Ring 1, Ring 2 and Ring 3, EAF refers to offshore points, EC to coastal points. Coordinates follow the MAGNA-SIRGAS/Colombia Bogotá Zone format (EPSG 3116).
