# Supplementary material for: Physicochemical water quality in coastal marine ecosystems: spatiotemporal variation between protected and disturbed areas
Source: PeerJ. 2026 Mar 19;14:e20855. doi: 10.7717/peerj.20855 (PMC13006004; doi:10.7717/peerj.20855)
Supplement: Supplemental Information 2 — Months with rainfall <100 mm were categorized as dry, while those with rainfall <100 mm were classified as rainy. [file peerj-14-20855-s002.docx]

| **Month-year** | **Average precipitation (mm) according to data from the IDEAM*** | **Category** |
| --- | --- | --- |
| Aprl-2021 | 125.04 | Rainy month |
| June-2021 | 266.07 | Rainy month |
| August-2021 | 471.77 | Rainy month |
| October-2021 | 307.67 | Rainy month |
| November-2021 | 77.50 | Dry month |
| December-2021 | 19.00 | Dry month |
| November-2022 | 301.81 | Rainy month |
| December-2022 | 35.41 | Dry month |
| February-2023 | 4.37 | Dry month |
| May-2023 | 126.30 | Rainy month |
| July-2023 | 177.20 | Rainy month |
| September-2023 | 149.04 | Rainy month |
| November-2023 | 227.54 | Rainy month |
| Mach-2024 | 45.19 | Dry month |

**Supplementary Table 2.** Classification of sampling months based on average monthly rainfall (mm), using data from IDEAM. Months with rainfall <100 mm were categorized as dry, while those with rainfall >100 mm were classified as rainy.
