# Supplementary material for: Physicochemical water quality in coastal marine ecosystems: spatiotemporal variation between protected and disturbed areas
Source: PeerJ. 2026 Mar 19;14:e20855. doi: 10.7717/peerj.20855 (PMC13006004; doi:10.7717/peerj.20855)
Supplement: Supplemental Information 3 [file peerj-14-20855-s003.docx]

**Supplementary Table 3.** Equipment, Technical Specifications, and Standards for Nutrient Analysis

| **Instrument** | **Technical specifications** |
| --- | --- |
| Dissolved oxygen meter | Dissolved oxygen meter 3310 set 1/ cellOx 325. Accuracy of the oxygen measurement ≤ 0.5 % of measured value ± 1digit, accuracy of the temperature measurement ≤ 0.1 k ± 1digit |
| pH meter | pH meter SI Analytics Handylab 100 equipped with a WTW Sentix 41 pH  electrode. Accuracy of the pH measurement ≤ 0.005 pH ± 1 digit. Accuracy of the temperature measurement ≤ 0.1 k ± 1digit  Accuracy of the voltage measurement ≤ 0.03mV± 1digit (-1200.0 +1200.0 mV)  ≤ 1mV± 1digit (-2500 + 2500mV) |
| Conductivity meter | Conductivity meter SI Analytics HandyLab 200. Accuracy of the conductivity mesasurement ≤ 0.5 % of measured value ± 1digit, accuracy of the temperature measurement ≤ 0.1 k ± 1digit |
| Thermo Scientific Genesys 180 UV/Visible Spectrophotometer | Light source: Xenon flash lamp |
|  | Spectral bandwidth 5 nm |
|  | Stray light <1.0% T @ 198 nm (KCl) |
|  | <0.05% T at 220 nm (Nal) |
|  | <0.03% T at 340 nm (NaNO2) |
|  | Wavelength accuracy ± 0.5 nm |
|  | Range 190 nm to 1100 nm |
|  | Resolution 0.2 nm, 0.5 nm, 1 nm, 2 nm, 5 nm |
| Ammonium standard solution | Traceable to SRM from NIST NH_4_Cl in H_2_O. 1000 mg/l NH_4_ Merck -Germany 1.19812.0500 |
| Nitrate standard solution | Traceable to SRM from NIST NaNO_3_ in H_2_O. 1000 mg/l NO_3_ Merck -Germany 1.19811.0500 |
| Nitrite standard solution | Traceable to SRM from NIST NaNO_2_ in H_2_O. 1000 mg/l NO_2_ Merck -Germany 1.19899.0500 |
| Phosphate standard solution | Traceable to SRM from NIST KH_2_PO_4_ in H_2_O. 1000 mg/l PO_4_ Merck -Germany 1.19898.0500 |
