# Supplementary material for: Physicochemical water quality in coastal marine ecosystems: spatiotemporal variation between protected and disturbed areas
Source: PeerJ. 2026 Mar 19;14:e20855. doi: 10.7717/peerj.20855 (PMC13006004; doi:10.7717/peerj.20855)
Supplement: Supplemental Information 4 — The regulated maximum seawater quality values for recreational use and for the conservation of marine biota, as well as the analytical detection method used. NTU = nephelometric units of turbidity. [file peerj-14-20855-s004.docx]

| **Variable/indicator** | **Unit** | **Maximum** | **Reference** | **Analytical detection method** | **Reference** |
| --- | --- | --- | --- | --- | --- |
| Total suspended solids -TSS | mg L^–1^ | 40 | Minambiente (2015) * | Gravimetric analysis, SM 2540-D | APHA, AWWA & WEF, 2017 |
| Turbidity | UNT | < 10 | Minambiente (2015) * | Nephelometric analysis, SM 2130-B | APHA, AWWA & WEF, 2017 |
| Fats and oils | mg L^–1^ | 5 | Minambiente (2015) * | Soxhlet extraction, SM 5520-D | APHA, AWWA & WEF, 2017 |
| Polycyclic aromatic hydrocarbons (PAHs | µg L^–1^ | 0.05 | Minambiente (2015) * | Liquid-liquid extraction with dichloromethane and fluorometric determination | Garay, Pinilla & Díaz, 2003 |
| Nitrates-NO_3_^–^ | µg L^–1^ | 95 | Alkhalidi, Al-Nasser, & Al-Sarawi (2022) | Colorimetric-Reduction Cd-Cu, SM 4500 – NO_3_^-^E | Strickland y Parsons (1972) |
| Nitrites-NO_2_^–^ | µg L^–1^ | 35 | Alkhalidi, Al-Nasser, & Al-Sarawi (2022) | Colorimetric sulfanilamide, SM 4500 – NO_2_^-^B | Strickland y Parsons (1972) |
| Ammonium -NH_4_^+^ | µg L^–1^ | 60 | Alkhalidi, Al-Nasser, & Al-Sarawi (2022) | Indo-fenol method, SM 4500 N C 4500 NH_4_^+^B-C | Strickland y Parsons (1972) |
| phosphates -PO_4_^3–^ | µg L^–1^ | 34 | Alkhalidi, Al-Nasser, & Al-Sarawi (2022) | Ascorbic acid method, SM 4500 – P E | Strickland y Parsons (1972) |
| Biochemical oxygen demand -BOD_5_ | mg L^–1^ O_2_ | 5 | Minambiente (2015) * | Incubation for five days, SM 5210 B AND 4500 – O C | APHA, AWWA & WEF, 2017 |
| Chemical oxygen demand -COD | mg L^–1^ O_2_ | 30 | Minambiente (2015) * | Photometric method, SM 5220-D | APHA, AWWA & WEF, 2017 |
| Thermotolerant coliforms | NMP/100 ml | 200 | Minambiente (2015) * | SM 9221 B | APHA, AWWA & WEF, 2017 |

**Supplementary Table 4.** Physicochemical variables assessed. The table shows the regulated maximum seawater quality values for recreational use and for the conservation of marine biota, as well as the analytical detection method used. NTU = nephelometric units of turbidity.

* Decree 1076 of 2015 of the Ministry of Environment and Sustainable Development for recreational marine waters and conservation of marine biota.
