# Supplementary material for: Physicochemical water quality in coastal marine ecosystems: spatiotemporal variation between protected and disturbed areas
Source: PeerJ. 2026 Mar 19;14:e20855. doi: 10.7717/peerj.20855 (PMC13006004; doi:10.7717/peerj.20855)
Supplement: Supplemental Information 5 [file peerj-14-20855-s005.docx]

**Supplementary Table 5**. Assessment Scale for the Marine Environmental Quality Index (ICAM)

| **Category** | **Quality scale** | **Description** |
| --- | --- | --- |
| **Optimal** | **100-90** | Excellent water quality |
| **Adequate** | **90-70** | Water with good conditions for aquatic life. |
| **Acceptable** | **70 -50** | Water in good condition and with few restrictions on use |
| **Inadequate** | **50-25** | Water that has many usage restrictions |
| **Very poor** | **25-0** | Waters with many restrictions that do not allow their proper use |
