# Supplementary material for: Physicochemical water quality in coastal marine ecosystems: spatiotemporal variation between protected and disturbed areas
Source: PeerJ. 2026 Mar 19;14:e20855. doi: 10.7717/peerj.20855 (PMC13006004; doi:10.7717/peerj.20855)
Supplement: Supplemental Information 6 — Correlations suggest no redundancy in between variables. [file peerj-14-20855-s006.docx]

**Supplementary Table S6.** Correlation analysis between physicochemical variables. Correlations suggest no redundancy in between variables.

|  | **TSS** | **DBO5** | **DQO** | **Turbidity** | **Oil/Fats** | **HAP** | **Nitrates** | **Nitrites** | **Ammonia** |
| --- | --- | --- | --- | --- | --- | --- | --- | --- | --- |
| **TSS** |  |  |  |  |  |  |  |  |  |
| **DBO5** | 0.602 |  |  |  |  |  |  |  |  |
| **DQO** | 0.327 | 0.272 |  |  |  |  |  |  |  |
| **Turbidity** | 5.960e-2 | 9.588e-2 | 0.283 |  |  |  |  |  |  |
| **Oil/Fats** | - | - | - | - |  |  |  |  |  |
| **HAP** | - | - | - | - | - |  |  |  |  |
| **Nitrates** | 0.877 | 0.553 | 0.356 | 0.121 | - | - |  |  |  |
| **Nitrites** | 0.543 | 0.404 | 0.391 | 0.656 | - | - | 0.548 |  |  |
| **Ammonia** | 6.051e-2 | 0.226 | 7.055e-2 | 0.414 | - | - | 0.149 | 0.172 |  |
| **Phosphates** | 5.375e-2 | 8.317e-2 | 0.240 | 0.800 | - | - | 0.176 | 0.525 | 0.744 |
