# Supplementary figures and images for: Physicochemical water quality in coastal marine ecosystems: spatiotemporal variation between protected and disturbed areas
Source: PeerJ. 2026 Mar 19;14:e20855. doi: 10.7717/peerj.20855 (PMC13006004; doi:10.7717/peerj.20855)

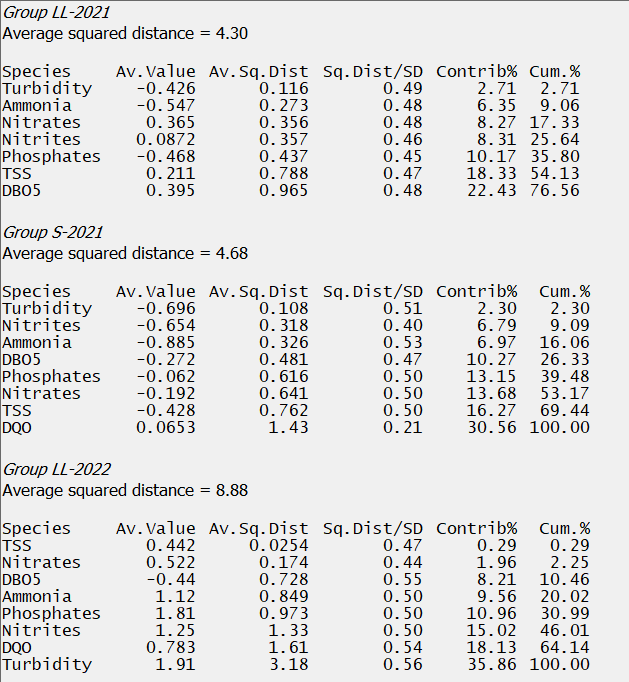
**Supplementary Table 7.** SIMPER analysis during the dry and rainy season 2021-2022

Supplement: Supplemental Information 7 [file peerj-14-20855-s007.docx]

**Supplementary Table 8.** SIMPER analysis during the dry and rainy season 2022-2024 and 2024


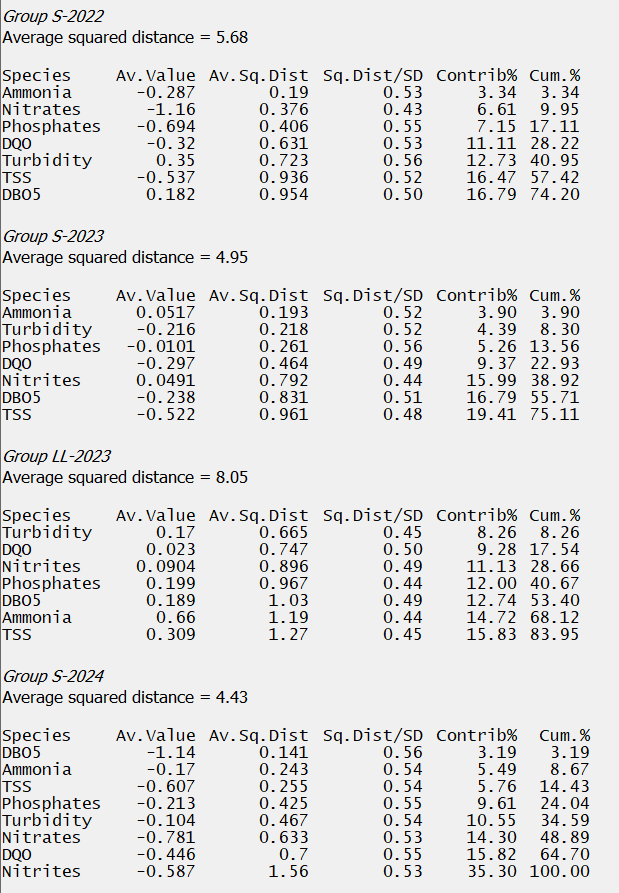


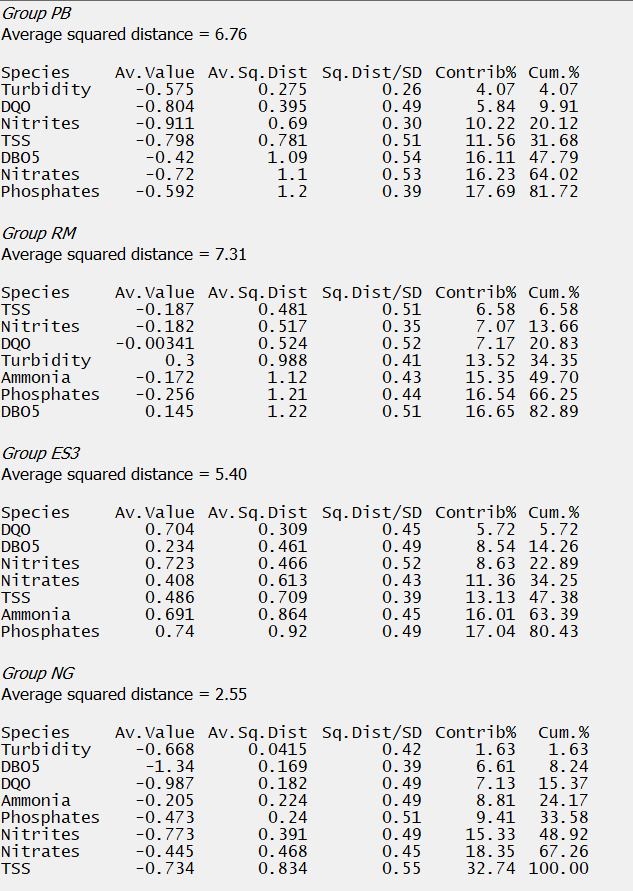


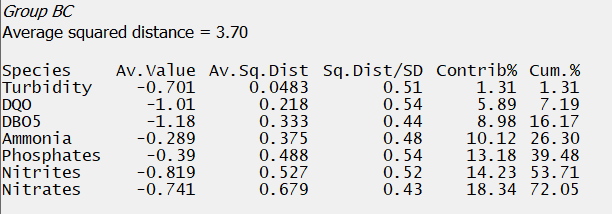

Supplement: Supplemental Information 8 [file peerj-14-20855-s008.docx]
