# Supplementary material for: Physicochemical water quality in coastal marine ecosystems: spatiotemporal variation between protected and disturbed areas
Source: PeerJ. 2026 Mar 19;14:e20855. doi: 10.7717/peerj.20855 (PMC13006004; doi:10.7717/peerj.20855)
Supplement: Supplemental Information 9 [file peerj-14-20855-s009.docx]

**Table S 9.** Eigen values of PCA.

| **PC** | **Eigenvalues** | **%Variation** | **Cum.%Variation** |
| --- | --- | --- | --- |
| **1** | 4.64 | 58.0 | 58.0 |
| **2** | 1.53 | 19.1 | 77.1 |
| **3** | 0.544 | 6.8 | 83.9 |
| **4** | 0.498 | 6.2 | 90.1 |
| **5** | 0.295 | 3.7 | 93.8 |
