# Supplementary material for: Physicochemical water quality in coastal marine ecosystems: spatiotemporal variation between protected and disturbed areas
Source: PeerJ. 2026 Mar 19;14:e20855. doi: 10.7717/peerj.20855 (PMC13006004; doi:10.7717/peerj.20855)
Supplement: Supplemental Information 10 [file peerj-14-20855-s010.docx]

**Supplementary Table 10.** COD/BOD ratio at sampling sites

| Sampling site | Date | COD | BOD | COD/BOD |
| --- | --- | --- | --- | --- |
| S1-2021-Apr-ES-R3 | Apr_2021 | 16,65 | 4,95 | 3,3631268 |
| S1-2021-Apr-ES-R12 | Apr_2021 | 15,92 | 5,73 | 2,7765424 |
| S1-2021-Apr-BS-R2 | Apr_2021 | 10,00 | 5,62 | 1,77830468 |
| S1-2021-Apr-IA-R2 | Apr_2021 | 10,00 | 2,18 | 4,58715596 |
| S1-2021-Apr-PB-R2 | Apr_2021 | 10,00 | 2,18 | 4,58715596 |
| S1-2021-Apr-RM-R2 | Apr_2021 | 13,59 | 3,71 | 3,66546763 |
| LL1-2021-Jun-ES-R3 | June_2021 | 18,68 | 5,60 | 3,33432481 |
| LL1-2021-Jun-ES-R12 | June_2021 | 23,94 | 6,42 | 3,73042462 |
| LL1-2021-Jun-BS-R2 | June_2021 | 13,71 | 5,27 | 2,59924147 |
| LL1-2021-Jun-IA-R2 | June_2021 | 10,00 | 2,18 | 4,58715596 |
| LL1-2021-Jun-PB-R2 | June_2021 | 10,00 | 2,18 | 4,58715596 |
| LL1-2021-Jun-RM-R2 | June_2021 | 13,65 | 2,18 | 6,25993884 |
| LL1-2021-Aug-ES-R2 | Aug_2021 | 30,42 | 7,96 | 3,81969863 |
| LL1-2021-Aug-ES-R11 | Aug_2021 | 29,39 | 7,74 | 3,79606007 |
| LL1-2021-Aug-BS-R1 | Aug_2021 | 16,48 | 6,37 | 2,58712716 |
| LL1-2021-Aug-IA-R1 | Aug_2021 | 13,10 | 3,76 | 3,48404255 |
| LL1-2021-Aug-PB-R1 | Aug_2021 | 19,03 | 6,77 | 2,81280788 |
| LL1-2021-Aug-RM-R1 | Aug_2021 | 25,06 | 8,93 | 2,80731889 |
| LL1-2021-Oct-ES-R3 | Oct_2021 | 26,96 | 6,95 | 3,87787724 |
| LL1-2021-Oct-ES-R12 | Oct_2021 | 24,13 | 7,12 | 3,39030388 |
| LL1-2021-Oct-BS-R2 | Oct_2021 | 15,59 | 6,08 | 2,56359649 |
| LL1-2021-Oct-IA-R2 | Oct_2021 | 13,03 | 3,29 | 3,96348884 |
| LL1-2021-Oct-PB-R2 | Oct_2021 | 12,57 | 5,95 | 2,11316527 |
| LL1-2021-Oct-RM-R2 | Oct_2021 | 22,02 | 9,08 | 2,42474302 |
| LL1-2021-Nov-ES-R3 | Nov_2021 | 48,83 | 3,99 | 12,2409903 |
| LL1-2021-Nov-ES-R12 | Nov_2021 | 19,58 | 5,18 | 3,77791174 |
| LL1-2021-Nov-BS-R2 | Nov_2021 | 15,03 | 4,52 | 3,3220339 |
| LL1-2021-Nov-IA-R2 | Nov_2021 | 13,76 | 2,18 | 6,31039755 |
| LL1-2021-Nov-PB-R2 | Nov_2021 | 16,47 | 4,67 | 3,52928571 |
| LL1-2021-Nov-RM-R2 | Nov_2021 | 24,88 | 5,06 | 4,91765481 |
| S2-2021-Dec-ES-R2 | Dec_2021 | 18,57 | 3,96 | 4,68470514 |
| S2-2021-Dec-ES-R11 | Dec_2021 | 19,36 | 5,11 | 3,78908735 |
| S2-2021-Dec-BS-R1 | Dec_2021 | 13,40 | 4,17 | 3,216 |
| S2-2021-Dec-IA-R1 | Dec_2021 | 10,00 | 2,18 | 4,58715596 |
| S2-2021-Dec-PB-R1 | Dec_2021 | 10,00 | 2,18 | 4,58715596 |
| S2-2021-Dec-RM-R1 | Dec_2021 | 22,90 | 4,21 | 5,4437401 |
| LL2-2022-Nov-IA-R2 | Nov_2022 | 10,83 | 2,18 | 4,96941896 |
| LL2-2022-Nov-RM-R2 | Nov_2022 | 27,63 | 5,78 | 4,77809798 |
| LL2-2022-Nov-PB-R2 | Nov_2022 | 14,10 | 2,18 | 6,46788991 |
| LL2-2022-Nov-ES-R4 | Nov_2022 | 35,39 | 5,21 | 6,78734117 |
| LL2-2022-Nov-ES-R9 | Nov_2022 | 33,12 | 4,83 | 6,85172414 |
| LL2-2022-Nov-ES-R14 | Nov_2022 | 35,93 | 4,38 | 8,20395738 |
| LL2-2022-Nov-NG-R2 | Nov_2022 | 14,17 | 2,18 | 6,49847095 |
| LL2-2022-Nov-BC-R2 | Nov_2022 | 14,40 | 2,18 | 6,60550459 |
| S3-2022-Dec-IA-R2 | Dec_2022 | 10,00 | 2,18 | 4,58715596 |
| S3-2022-Dec-RM-R2 | Dec_2022 | 18,30 | 5,87 | 3,11697899 |
| S3-2022-Dec-PB-R2 | Dec_2022 | 13,04 | 5,51 | 2,36864407 |
| S3-2022-Dec-ES-R3 | Dec_2022 | 20,58 | 6,96 | 2,95572044 |
| S3-2022-Dec-ES-R8 | Dec_2022 | 18,49 | 6,91 | 2,67518688 |
| S3-2022-Dec-ES-R13 | Dec_2022 | 20,89 | 5,38 | 3,88252788 |
| S3-2022-Dec-NG-R1 | Dec_2022 | 11,90 | 2,18 | 5,45718654 |
| S3-2022-Dec-BC-R1 | Dec_2022 | 10,00 | 3,63 | 2,75229358 |
| S3-2023-Feb-IA-R1 | Feb_2023 | 10,00 | 2,18 | 4,58715596 |
| S3-2023-Feb-RM-R1 | Feb_2023 | 15,27 | 4,55 | 3,35531136 |
| S3-2023-Feb-PB-R1 | Feb_2023 | 13,59 | 4,52 | 3,00589971 |
| S3-2023-Feb-ES-R2 | Feb_2023 | 22,04 | 6,00 | 3,67454141 |
| S3-2023-Feb-ES-R7 | Feb_2023 | 17,74 | 5,13 | 3,4604878 |
| S3-2023-Feb-ES-R12 | Feb_2023 | 19,29 | 5,00 | 3,8625292 |
| S3-2023-Feb-NG-R2 | Feb_2023 | 12,40 | 2,18 | 5,68654434 |
| S3-2023-Feb-BC-R2 | Feb_2023 | 13,66 | 2,18 | 6,26452599 |
| LL3-2023-May-IA-R2 | May_2023 | 13,21 | 4,10 | 3,22113821 |
| LL3-2023-May-RM-R2 | May_2023 | 16,81 | 5,56 | 3,02519496 |
| LL3-2023-May-PB-R2 | May_2023 | 18,25 | 4,14 | 4,40901771 |
| LL3-2023-May-ES-R3 | May_2023 | 24,00 | 7,27 | 3,30018332 |
| LL3-2023-May-ES-R8 | May_2023 | 21,13 | 5,91 | 3,57747671 |
| LL3-2023-May-ES-R16 | May_2023 | 21,64 | 6,31 | 3,42884311 |
| LL3-2023-May-NG-R1 | May_2023 | 14,96 | 2,18 | 6,86085627 |
| LL3-2023-May-BC-R1 | May_2023 | 15,77 | 2,18 | 7,23394495 |
| LL3-2023-Jul-IA-R1 | July_2023 | 10,00 | 2,18 | 4,58715596 |
| LL3-2023-Jul-RM-R1 | July_2023 | 12,56 | 2,18 | 5,76299694 |
| LL3-2023-Jul-PB-R2 | July_2023 | 12,18 | 2,18 | 5,58562691 |
| LL3-2023-Jul-ES-R4 | July_2023 | 22,77 | 5,08 | 4,48195538 |
| LL3-2023-Jul-ES-R9 | July_2023 | 26,82 | 4,64 | 5,7808908 |
| LL3-2023-Jul-ES-R17 | July_2023 | 25,56 | 4,31 | 5,92465224 |
| LL3-2023-Jul-NG-R2 | July_2023 | 10,80 | 2,60 | 4,1525641 |
| LL3-2023-Jul-BC-R2 | July_2023 | 10,92 | 3,23 | 3,37628866 |
| LL3-2023-Sept-IA-R2 | Sept_2023 | 14,63 | 5,48 | 2,6674772 |
| LL3-2023-Sept-RM-R2 | Sept_2023 | 17,73 | 5,58 | 3,17872086 |
| LL3-2023-Sept-PB-R2 | Sept_2023 | 19,16 | 5,14 | 3,73069435 |
| LL3-2023-Sept-ES-R3 | Sept_2023 | 27,54 | 8,31 | 3,31540931 |
| LL3-2023-Sept-ES-R8 | Sept_2023 | 27,37 | 8,81 | 3,10787282 |
| LL3-2023-Sept-ES-R13 | Sept_2023 | 27,16 | 7,26 | 3,74408454 |
| LL3-2023-Sept-NG-R2 | Sept_2023 | 14,35 | 3,78 | 3,80052957 |
| LL3-2023-Sept-BC-R3 | Sept_2023 | 13,05 | 3,72 | 3,50896057 |
| LL3-2023-Nov-IA-R3 | Nov_2023 | 13,13 | 4,82 | 2,7266436 |
| LL3-2023-Nov-RM-R3 | Nov_2023 | 16,17 | 9,36 | 1,72659309 |
| LL3-2023-Nov-PB-R3 | Nov_2023 | 11,36 | 7,65 | 1,48605057 |
| LL3-2023-Nov-ES-R4 | Nov_2023 | 18,85 | 6,83 | 2,76190476 |
| LL3-2023-Nov-ES-R9 | Nov_2023 | 17,28 | 6,04 | 2,86305908 |
| LL3-2023-Nov-ES-R14 | Nov_2023 | 21,10 | 6,28 | 3,36175299 |
| LL3-2023-Nov-NG-R3 | Nov_2023 | 12,03 | 2,18 | 5,51987768 |
| LL3-2023-Nov-BC-R3 | Nov_2023 | 12,20 | 2,18 | 5,59633028 |
| S4-2024-Mar-IA-R3 | Mar_2024 | 10,00 | 2,18 | 4,58715596 |
| S4-2024-Mar-RM-R3 | Mar_2024 | 14,90 | 2,18 | 6,83486239 |
| S4-2024-Mar-PB-R3 | Mar_2024 | 11,97 | 2,18 | 5,48929664 |
| S4-2024-Mar-ES-R3 | Mar_2024 | 21,53 | 3,18 | 6,77503933 |
| S4-2024-Mar-ES-R9 | Mar_2024 | 17,45 | 3,26 | 5,35549872 |
| S4-2024-Mar-ES-R15 | Mar_2024 | 20,35 | 2,93 | 6,94934548 |
| S4-2024-Mar-NG-R2 | Mar_2024 | 10,00 | 2,18 | 4,58715596 |
| S4-2024-Mar-BC-R2 | Mar_2024 | 10,00 | 2,18 | 4,58715596 |
