# Supplementary material for: Physicochemical water quality in coastal marine ecosystems: spatiotemporal variation between protected and disturbed areas
Source: PeerJ. 2026 Mar 19;14:e20855. doi: 10.7717/peerj.20855 (PMC13006004; doi:10.7717/peerj.20855)

**Supplementary Table 11.** Calculation of the marine environmental quality index (ICAM) at sampling stations during dry and rainy periods

| **Site** | **Seasons** | **mean_ICAM** | **sd_ICAM** | **ICAM_X_SD** |
| --- | --- | --- | --- | --- |
| NG | Dry | 79,20479 | 14,300334 | 79.2 ± 14.3 |
| NG | Rainy | 59,9195 | 6,884009 | 59.92 ± 6.88 |
| BC | Dry | 72,88178 | 2,595432 | 72.88 ± 2.6 |
| BC | Rainy | 55,2715 | 9,77903 | 55.27 ± 9.78 |
| IA | Dry | 69,54364 | 12,844783 | 69.54 ± 12.84 |
| IA | Rainy | 53,46363 | 9,780731 | 53.46 ± 9.78 |
| ES | Dry | 38,56953 | 12,727065 | 38.57 ± 12.73 |
| ES | Rainy | 19,29199 | 11,292047 | 19.29 ± 11.29 |
| RM | Dry | 42,51178 | 6,561224 | 42.51 ± 6.56 |
| RM | Rainy | 32,4789 | 23,205065 | 32.48 ± 23.21 |
| PB | Dry | 48,92206 | 7,570407 | 48.92 ± 7.57 |
| PB | Rainy | 37,77233 | 23,701114 | 37.77 ± 23.7 |


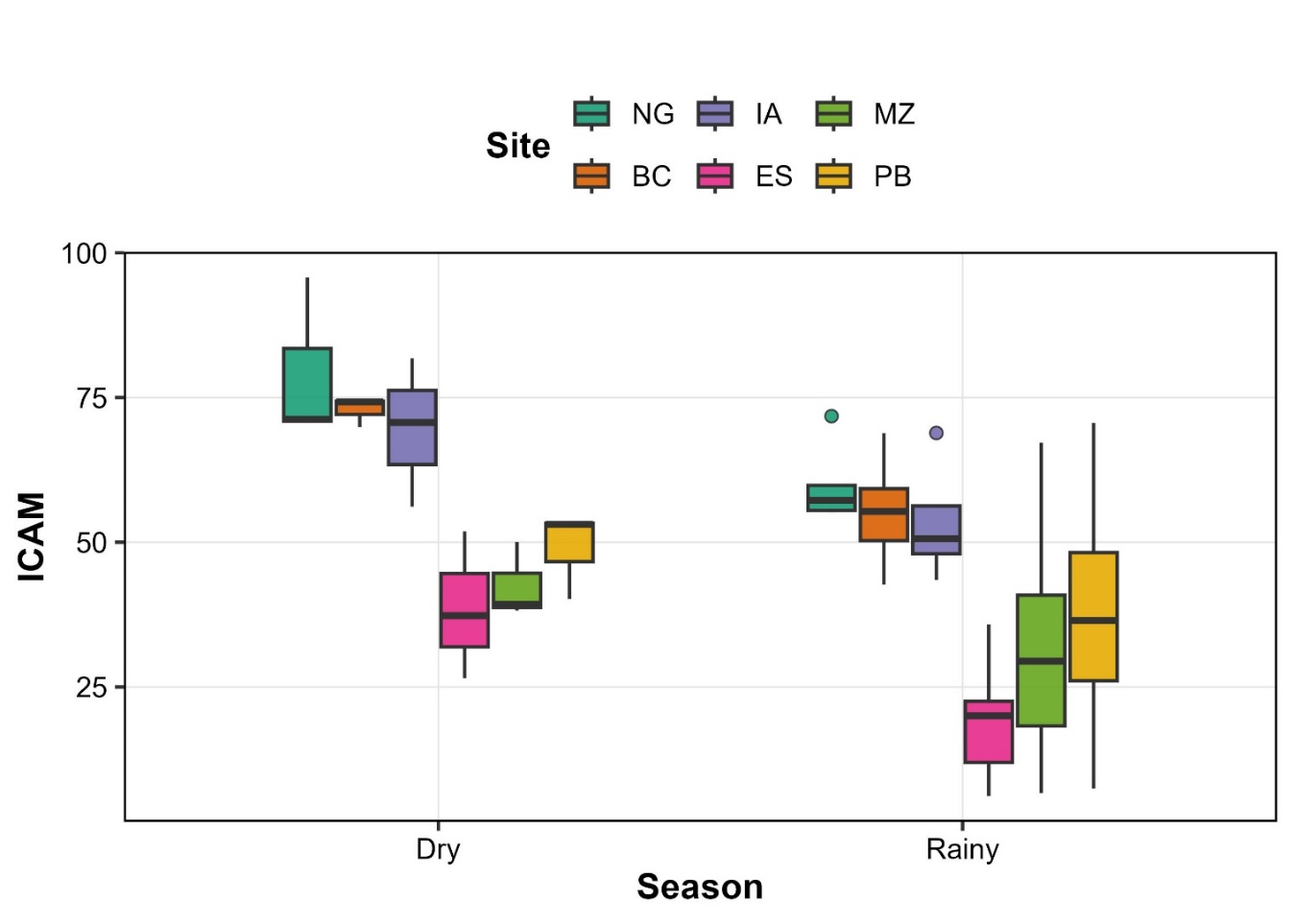

Supplement: Supplemental Information 11 [file peerj-14-20855-s011.docx]
